# Shining Light on Halide Perovskites: Teaching Analytical Chemistry Using Flexible, Inquiry-Based Experiments

**DOI:** 10.1021/acs.jchemed.5c00906

**Published:** 2026-02-19

**Authors:** Kristel M. Forlano, Eliana Bernat, Pamela Doolittle, Dominic Colosi, Song Jin, Amanda Rae Buchberger

**Affiliations:** Department of Chemistry, 5228 University of Wisconsin—Madison, Madison, Wisconsin 53706, United States

**Keywords:** Analytical Chemistry, Inquiry-based/Discovery Learning, Second-year Undergraduate, Materials Science, Semiconductors, Spectroscopy, Synthesis

## Abstract

Two-dimensional (2D) metal halide perovskites are promising next generation semiconducting materials at the forefront of research in solar cells, LEDs, and other devices. Here, we report on an undergraduate intermediate analytical chemistry laboratory experience where students were taught fundamental chemistry concepts, including solubility, complexation, spectroscopy, and microscopy, through the introduction and study of 2D halide perovskite materials. Students explore multiple facets of perovskite synthesis, structure, and properties through a modular set of experiments that students used to form a holistic picture of this material. Importantly, this inquiry-based lab supports students through a guided research process, and students report high interest and learning gains from an end of the semester survey. We further discuss ways to adapt this lab to course, student, equipment, and budget needs. Overall, this laboratory experience teaches and applies the fundamental concepts and tools of analytical chemistry to the contemporary materials research field.

## Graphical abstract

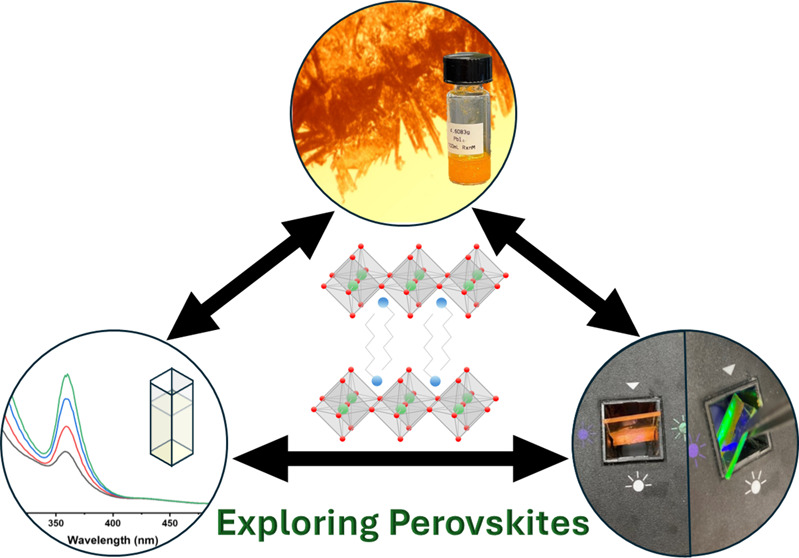

Metal halide perovskites are solution-processable semiconductor materials promising for applications in solar cells, LEDs, and other devices.^1–3^ Three-dimensional (3D) halide perovskites refer to a class of crystal structures with the formula ABX_3_, where A is a small monovalent cation, B is a divalent metal, and X is a halide. The structure is made up of corner-sharing BX_6_ octahedra with the A-cation sitting in the middle of the pockets or cages made by the BX_6_ octahedra network (Figure 1A). From the 3D perovskite, two-dimensional (2D) or quasi-2D layers can form through the incorporation of a large organic cation (LA), generally with an ammonium headgroup: (LA)_*m*_(A)_*n*‑1_B_*n*_X_3*n*+1_. The number of BX_6_ octahedra layers between a layer of the organic “spacer cation” is denoted through *n*, where *n* = 1 is one layer of BX_6_ octahedra, *n* = 2 is two layers of BX_6_ octahedra, *etc*. and *m* denotes whether there will be a bilayer of a monoammonium spacer cation or a monolayer of a diammonium spacer cation (Figure 1A).^4,5^ The perovskite materials are highly tunable and easy to synthesize in solutions, which lends them the ability to be readily explored within an undergraduate classroom (see variations used in this lab in Figure 1B).

**Figure 1:**
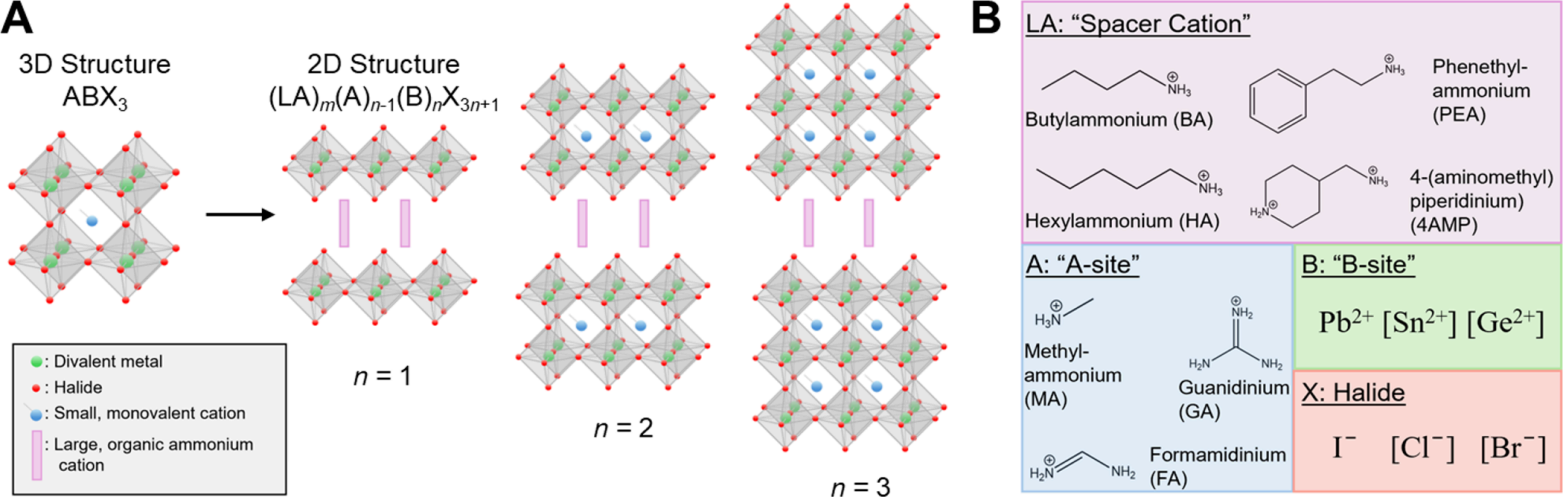
A) Crystal structures of 3D perovskite with the formula ABX_3_ and 2D perovskites (LA)_*m*_(A)_*n*‑1_B_*n*_X_3*n*+1_ for layer number *n* = 1, 2, and 3. B) Options for tuning the perovskite crystal structure by different sites: LA, A, B, and X. Compounds in brackets show options for perovskite crystal structure not utilized in this lab. For more extensive discussion on the 2D perovskite structure and how the structure influences the semiconductor properties, see Supporting Information Notes for Instructors and Discussion Activity for Part 2.

Despite their prominence in contemporary materials research, few reports exist on incorporating halide perovskites into undergraduate curricula, with current reports focusing on 3D perovskite and perovskite quantum dots and none to our knowledge discussing 2D perovskites.^6–12^ In contrast to 3D perovskites, 2D perovskites are more stable against environmental degradation from oxygen and water due to the hydrophobic nature of the organic spacer cation having a protective role on the perovskite surface.^13–15^ In addition, 2D perovskites have more structural tunability, which influences their semiconductor properties.^16–20^ For these reasons, 2D perovskites are of high interest to the research community, and that is why they were chosen as the focus of this laboratory experiment.

Bringing cutting edge research and research experiences to the undergraduate laboratory setting is an exciting curriculum field, as shown by the explosion of popularity of inquiry-based learning (IBL) laboratory experiences and course based undergraduate research experiences (CUREs).^21–24^ IBL breaks down further into project-based learning (PBL), where students are expected to tackle real-world, open-ended, student-drive research questions.^24–26^ According to the Buck Institute for Education, PBL units require (1) authenticity, (2) academic rigor, (3) application of learning through teams and communicating and analyzing data, (4) active exploration, (5) interactions of learners, and (6) formal and informal assessment practices.^27^ Especially within the analytical chemistry classroom, PBL is often implemented,^28^ but integrating material science (such as perovskites) is uncommon or nonexistent, showing a clear gap.

As such, we designed a semester-long PBL lab experience to introduce students in an undergraduate intermediate analytical chemistry class to the active materials research field of halide perovskite semiconductors, while encompassing the instruction of core course content and fundamental chemistry concepts. We consider this lab to be Open Inquiry, according to the levels of inquiry proposed by Buck et al.,^29,30^ where students were guided through designing their own experiments to answer questions about the system that they were examining.^28^ This lab experience fits into the traditional analytical chemistry curriculum by covering core concepts of UV–vis absorbance spectroscopy, solubility equilibria, complexation, and fluorescence spectroscopy, as well as techniques not traditionally covered in an analytical chemistry curriculum, such as thin film spectroscopy and optical microscopy.^31^ We also discuss additional techniques, such as inductively coupled plasma (ICP), that can be added into this lab. The multitude of synthesis and characterization techniques for the materials described here allow for the flexible adaptation of this lab to varying university resources and different courses beyond analytical chemistry, such as inorganic or physical chemistry or a material science course. This experiment was originally piloted in the Spring 2024 semester, and the following report describes an updated and modified experience implemented in the Spring 2025 semester. A postexperiment survey was conducted at the end of the semester for students to provide feedback and self-report skill gains. This study falls under the umbrella of program assessment; therefore, no IRB approval was required.

## Experimental Overview

### Learning Objectives (LOs)

The goal in designing this laboratory experience was to teach fundamental analytical chemistry concepts through applications in the field of materials science and perovskites (Figure 2). By the end of this lab, students are expected to

1) Describe how Pb complexation chemistry allows for measuring Pb concentration using a spectrophotometry method, and how solvent choice affects solubility.
2) Optimize the synthesis of a variety of 2D perovskite crystals and characterize the products through optical microscopy.
3) Use UV–vis spectrophotometry to measure and quantitatively compare the concentrations of leftover Pb in different perovskite precursor solutions. Propose a relationship between solvent choice, solubility, and perovskite crystal growth.
4) Design, modify, and test instrumental and experimental parameters for measuring optical properties of perovskite crystals, such as transmittance and photoluminescence. Connect measured optical properties to the expected perovskite crystal structure.
5) Scientifically communicate via a final presentation the results of their experiments.

**Figure 2:**
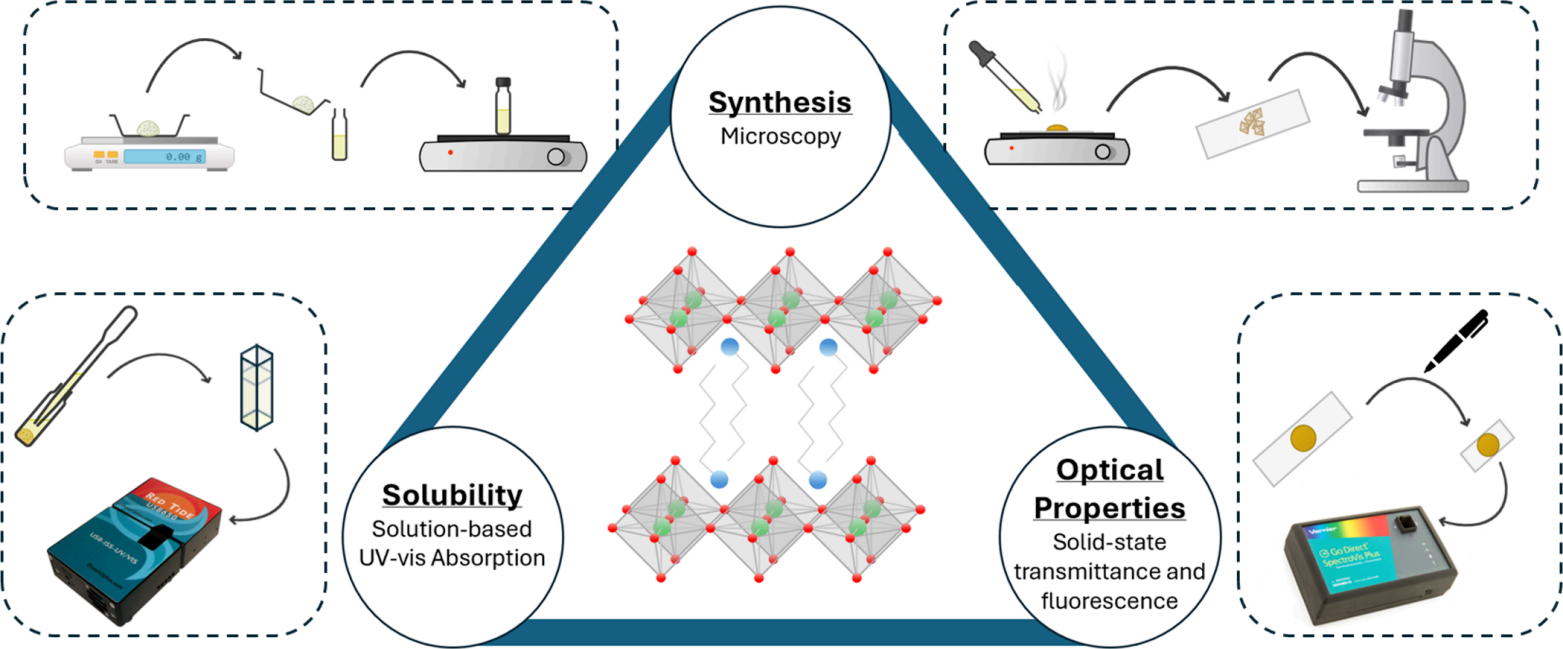
Schematic illustration of the learning goals of the project lab and analytical techniques taught, with cartoon schematics of the various experimental set ups.

### Structure of the Lab Course

This lab experience took place in an undergraduate analytical chemistry course in Spring 2025 with 40 participants. Completion of general chemistry is a prerequisite for analytical chemistry, but no other courses are required. Some students may have taken organic chemistry prior to this course. Students were in their first (*n* = 18), second (*n* = 18), or third (*n* = 4) year at UW-Madison and majoring in chemical engineering (*n* = 17), chemistry (*n* = 12), biochemistry (*n* = 9), environmental engineering (*n* = 1), or undecided (*n* = 1). The lab experience began in the third week and was separated into Parts 1 and 2 that spanned nearly the entire semester noncontinuously (Figure 3). In this course, students attend lab twice a week for 4 h each, a discussion (or recitation) class for 50 min once a week, and lecture for 50 min twice a week. All 40 students were in the same lecture together, but lab was split across 3 sections, with a Teaching Assistant assigned to each, although the 3 sections took place at the same time and in the same lab space. Lecture focused on conceptual topics in analytical chemistry that may or may not have been utilized in this lab; only one lecture was used to introduce concepts directly related to perovskites (in the middle of Week 8, prior to starting Part 2 preparation or planning). Students were assigned to groups of 3–4 (which resulted in 13 groups total for Spring 2025) and began working together near the start of the semester (i.e., Week 3, prior to the “Group Charter” activity). In discussion, students completed a lab-specific preparation activity (for each part the week before the lab days started) that was designed to introduce students to the theoretical concepts to be applied in the lab (see for handouts). Students had four lab days to complete each part of the project, with additional in-lab time budgeted for data analysis and preparation of final deliverables—a written report for Part 1 and a cumulative group presentation following Part 2. Final presentations took place the week before finals. See the Instructor Notes for the full schedule of the course, which also shows how other elements of the course, such as standard laboratories covering analytical chemistry topics not included in this experience, were interspersed.

**Figure 3:**
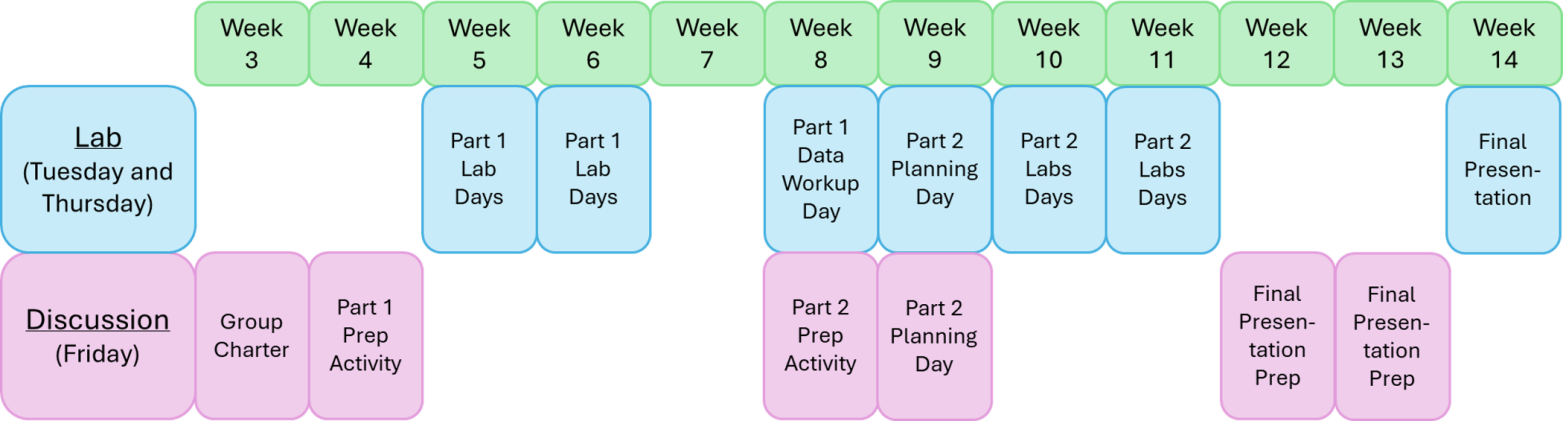
Schematic of the lab course structure showing the timeline of discussion and laboratory activities pertaining to this laboratory over the course of a semester. Weeks 1 and 2 had no project lab activities for the students. Other open areas contained standard laboratories and activities pertaining to the rest of the course material.

### Chemicals and Equipment

The perovskite precursors used, as shown in Figure 1B, were *n*-hexylammonium iodide (C_6_H_11_NH_3_I, HAI), *n*-butylammonium iodide (C_5_H_9_NH_3_I, BAI), phenethylammonium iodide (C_8_H_9_NH_3_I, PEAI), 4-(aminomethyl)­piperidine (C_6_H_14_N_2_, 4AMP), methylammonium iodide (CH_3_NH_3_I, MAI), formamidinium iodide (CH_5_N_2_I, FAI), guanidinium iodide (CH_6_N_3_I, GAI), and lead iodide (PbI_2_). The solvents used were 57 wt % hydroiodic acid (HI), 50 wt % hypophosphorous acid (H_3_PO_2_), dimethylformamide (DMF), and deionized water. Major equipment used included a hot plate, a spectrophotometer that is capable of near-UV–vis (200–400 nm) detection, a spectrophotometer that is capable of fluorescence (<510 nm excitation source) and UV–vis (400–800 nm) detection, and an optical microscope with an attached USB digital camera (and appropriate software to take videos, photos, *etc*.; example: Swift microscope). Additional supplies used include glass slides, glass scoring tools, UV flashlights (5W, 395 nm), wiretrols (5–100 μL), 2-dram vials, and quartz cuvettes.

### Part 1 Experimental Description Overview

In Part 1 of the project lab, students explored and compared the solubility of PbI_2_ in acidic media and water. The acid solvent used was a 1:1 v/v mix of HI and H_3_PO_2_ (the H_3_PO_2_ helps to stabilize HI against oxidation). From this point forward, any discussion of HI references this 1:1 mixture of the acids. Lead forms coordination complexes with iodide in solution which are visible through UV–vis spectrophotometry.^32,33^ The [PbI_3_]^−^ complex is typically the only one observed under the conditions used, with a maximum absorbance at approximately 360 nm, and was used as an approximation for the total Pb concentration. Solutions of PbI_2_ in HI with known concentrations were made, the absorption measured, and a calibration curve created following Beer’s Law. Because the [PbI_3_]^−^ complex is strongly absorbing, the optimal concentration range for standard solutions was 10–100 μM for our equipment.

Each student group was assigned the task of examining the solubility of PbI_2_ in solutions with different concentrations of HI in water. From the original acid mixture, instructors made additional solutions that were 75:25, 50:50, and 25:75 HI:H_2_O. Students were given vials for the different solvent ratios that were visibly saturated with PbI_2_. By measurement of the concentration of [PbI_3_]^−^ in the solution, the PbI_2_ concentration (and thus its total solubility) can be inferred. Serial dilutions are needed to get the concentration of the saturated samples within the range of the created calibration curve. Data from all groups was pooled, and students could perform statistical analysis and determine a trend in solubility across the entire class’s data.

### Part 2 Experimental Description Overview

Part 2 of the project lab began with the synthesis of perovskite crystals. Students were first given a basic procedure to synthesize (HA)_2_PbI_4_ (*n* = 1) as a model system due to the ease of synthesis for this perovskite. Then, students were asked to pick a variable in perovskite composition to explore, where they had to design their own experimental procedures. Students explored 3 different facets of the synthesized perovskites:

a) The crystals were synthesized in both HI and DMF from the ammonium iodide precursor salts. Photos of the crystals in the vials and under a microscope are collected and compared to show differences in *crystal growth and morphology* between different perovskites and solvents. If ultraviolet light from a UV flashlight is shined on the perovskite crystal, photoluminescence at the approximate energy of the bandgap can often be observed.
b) Using the same method developed in Part 1, students explored the difference in *solubility* of the perovskites through measurement of the soluble lead concentration and comparison across the two solvents and their chosen design variable.
c) The optical properties of the semiconducting perovskite crystals are examined through spectroscopy. UV–vis spectrophotometers that measure solid samples are more specialized than cuvette-based instruments to probe solutions, but thin film-like solid samples can be measured in a cuvette-based spectrometer after some development efforts. Students used thin film samples drop cast on glass slides to measure percent *transmittance and fluorescence* of various perovskites and connected the measurements to the bandgap of each perovskite.

## Hazards

PbI_2_ exists as a powder, making it dangerous to inhale, and should always be handled in a fume hood, with the option of using a respirator. To mitigate this risk for students, PbI_2_ was always distributed in solutions (0.1, 0.3, or 0.5 M) made by the instructional staff. However, lead-based solutions are still very hazardous if in contact with skin or eyes or ingested. Therefore, gloves, lab coats, safety goggles, pants, and close-toed shoes were always required while working in lab. Lead waste should be disposed of through proper chemical disposal channels that can handle heavy metal processing.

HI and H_3_PO_2_ are acidic and can cause severe skin and eye irritation. DMF shares these risks but can also readily permeate standard gloves and skin while carrying dissolved ions with it. As DMF is handled with lead dissolved in it for much of this lab, if any DMF solution gets on gloves, the gloves should immediately be replaced. Any remaining solutions of HI:H_3_PO_2_ need to be neutralized prior to disposal. Since perovskites are formed via a heating and cooling process, HI should not be heated above the boiling point (127 °C). The perovskite solutions in sealed vials can be handled outside of a fume hood, as well as brief open handling (e.g., taking an absorbance measurement in a cuvette). Drop cast films should be made inside the fume hood to allow for solvents to evaporate safely.

When cutting glass slides by scoring with a diamond tipped pen and breaking along the line, caution should be taken to avoid holding the glass slide too close to the score line while breaking, to avoid stabbing by the glass. The cut slides should not be handled by the broken edges. This technique should be demonstrated first by instructors and supervised a few times before students are allowed to do it on their own.

## Results and Discussion

### Part 1: Solubility of PbI_2_ in Water vs Acid

PbI_2_ is a well-known example of an insoluble compound in water (K_sp_ = 9.8 × 10^–9^). However, in HI its solubility greatly increases, which is attributed to the stepwise formation of lead-iodide complexes. When dissolved in solution with excess iodide, Pb^2+^ undergoes a series of equilibria forming successive complexes: [PbI]^+^, [PbI_2_], [PbI_3_]^−^, and [PbI_4_]^2–^.^34^ A thorough discussion of this equilibrium process can be found in the Part 1 Discussion Activity for students in the Supporting Information. These lead iodide complexes can be observed through UV–vis absorption spectroscopy.^33^ Students were tasked with creating standards of PbI_2_ dissolved in the HI solution in the concentration range of ∼10–100 μM through serial dilutions of a 0.1 M PbI_2_ stock solution provided to them. The absorbance was measured, and a prominent peak was observed at ∼360 nm that is commonly attributed to the [PbI_3_]^−^ complex (Figure 4A). Since only one absorbance peak was observable, it was assumed that all dissolved lead was in the [PbI_3_]^−^ complex, and its concentration is therefore a proxy for total lead dissolved in solution. A calibration curve made from the maximum absorbance of the 360 nm peak shows good linearity (Figure 4B).

**Figure 4:**
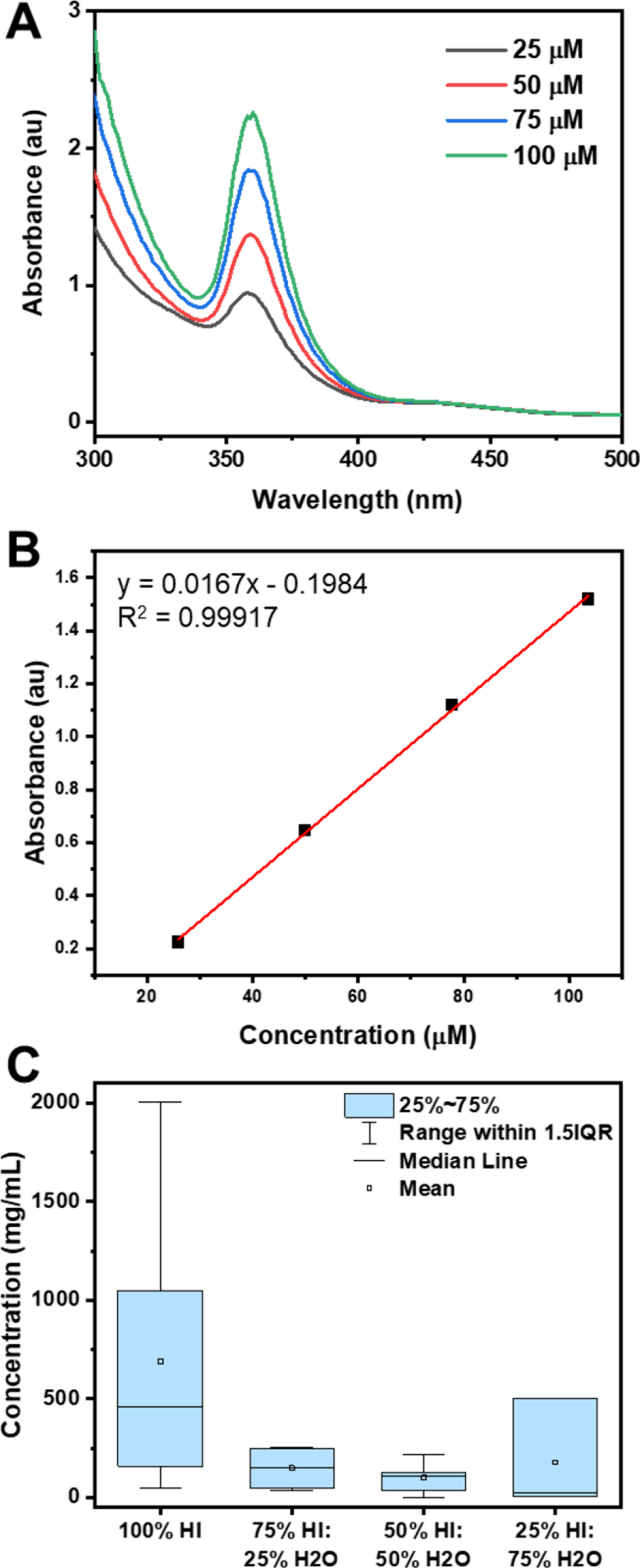
A) Absorbance spectra for different concentrations of PbI_2_ in HI/H_3_PO_2_ and B) corresponding calibration curve from the maximum absorbance at 360 nm. At 360 nm, a linear relationship between concentration and absorbance can be gleaned to allow quantitation of [PbI_3_]^−^ and infer Pb concentration in solution. C) Summary of class data for determination of Pb concentration in solution for different ratios of HI:H_2_O. Overall trend shows decreasing solubility of PbI_2_ with increasing H_2_O (IQR: Interquartile range). Student ANOVA results found a statistical difference in the solubility across all solvents.

The maximum solubility of PbI_2_ in water under standard conditions is 0.578 mg/mL.^34^ To show how the solubility of PbI_2_ changes with HI concentration, students calculated the maximum solubility of PbI_2_ in HI and then in various mixtures of HI/H_2_O. To do this, students were given a vial of solution saturated with PbI_2_. As the initial concentration of the saturated solution was unknown, students had to test many diluted samples to get within the range of their calibration curve. The calculated solubility results from all student groups in the class (obtained via submission on a shared Excel sheet) were pooled, and students were able to see (and statistically test via ANOVA) how the solubility of PbI_2_ decreases as the amount of water increases (Figure 4C). We believe that the variability in student results seen in the box plot is due to not filtering out solid PbI_2_ in the saturated solutions before measurement. This is a change that will be implemented in future iterations of this lab. Of note, the variation can be used by instructors as a conversation point to discuss the importance of careful lab skills and critical interpretation of the data with the students.

### Part 2a: Synthesis and Microscopy Imaging of Perovskite Crystals

After exploring how PbI_2_ solubility depends on solvents and complexation equilibria in Part 1, students next investigated how different experimental parameters influenced the growth of 2D perovskites. The general solution-based synthesis of 2D perovskites is straightforward and well reported by the perovskite research community.^5,35,36^ The precursor salts in stoichiometric amounts were added to a vial with a solvent (HI or DMF), heated to approximately 100 °C (when most if not all precursor salts should dissolve) before being allowed to cool (approximately 10 °C every 10 min). One possible set up is shown in Figure 5A, where a water bath is used to control heating. Other alternatives include placing the vial directly on a hot plate or putting the vial in an oven. This heating and cooling process is very flexible and tolerant. The faster solutions are cooled, the more numerous, but smaller, crystals will form, while larger crystals will grow with slower cooling. All student groups made (HA)_2_PbI_4_ as a practice example and control using precursor salts HAI and PbI_2_ in a 2:1 molar ratio. A good starting concentration for the final perovskite crystal is 0.1 M, which can be adjusted based on perovskite solubility.

**Figure 5:**
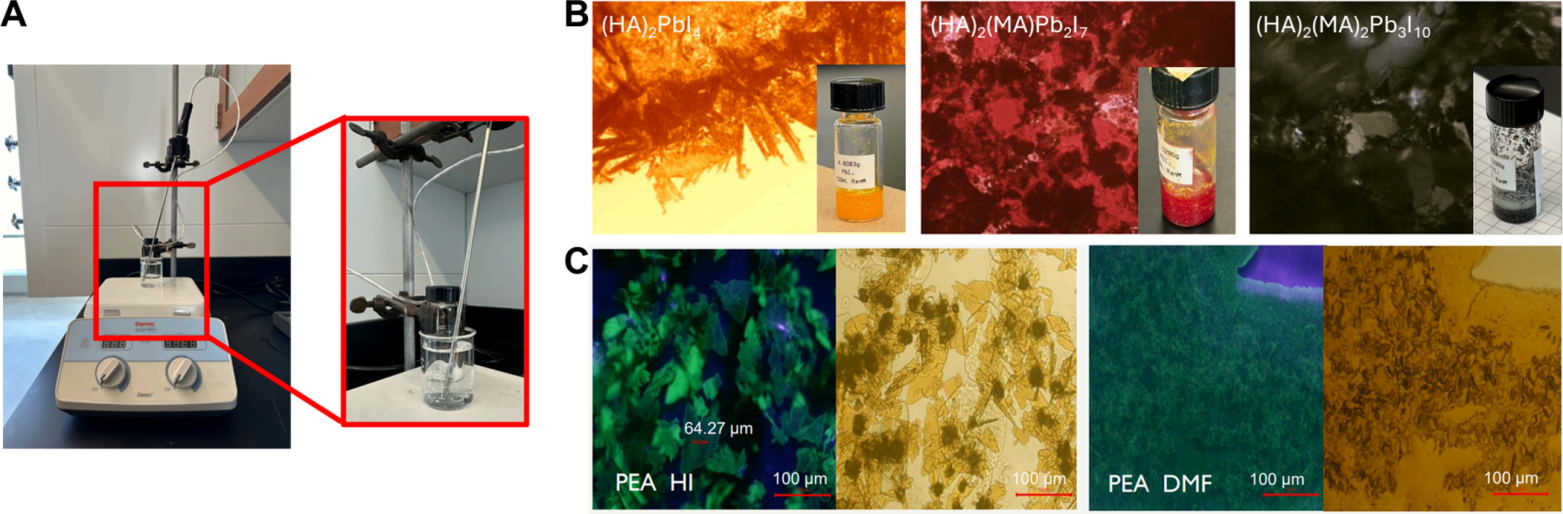
A) Possible set up for perovskite synthesis, using a water bath on a hot plate. While not necessary, the hot plate set up includes a temperature probe to allow precise control of the solution temperature. B) Optical microscopy images of (HA)_2_(MA)_*n*‑1_Pb_*n*_I_3*n*+1_ perovskite crystals at room temperature showing the typical colors for *n* = 1 (orange), *n* = 2 (red), and *n* = 3 (black) lead iodide perovskites with insets showing photographs of crystals in vials. Data was generated by the student group Reflux and Chill, which included Ashley F. and 2 other students. C) Fluorescence and white light microscopic images of (PEA)_2_PbI_4_ showing a difference in crystal morphology between HI and DMF solvents. Data was generated by the student group Perovskite Princesses, which included Jiyoung Amanda H. and 2 other students.

2D lead perovskite crystals exhibit different colors depending on the composition, particularly the *n* number. Figure 5B shows the typical orange, red, and black colors for *n* = 1, 2, and 3 lead iodide perovskite crystals, respectively.^4,5,35^ The crystal color is a very good initial indicator of what *n* phase was formed. While stoichiometric amounts of the precursors can be a good starting point, often the recipes for making perovskite crystals need to be tuned. In order to drive the crystal formation to a certain composition, Le Chatelier’s Principle can be applied. If higher *n* phase crystals are desired, more lead and A-cation precursors can be added (or less spacer cation). The opposite can be applied to drive to a lower *n* phase.

Each student group had a chance to choose a variable in perovskite synthesis to explore (all options are shown in Figure 1B). Choice 1 explored the synthesis of perovskites across changing the spacer cation, all in *n* = 1 perovskites.^16^ Here, students were given the precursor salts BAI, PEAI, and 4AMP, in addition to the control of HAI. The differing molecular structures of these spacer cations induce different perovskite solubilities. Choice 2 explored *n* = 2 crystals with changing the A-cation (MA, FA, and GA) in (HA)_2_(A)­Pb_2_I_7_ crystals.^37,38^ Students will notice that driving the formation of *n* = 2 perovskites differs depending on the A cation, with GA (the largest) being the most difficult. Choice 3 explored the *n* = 1, 2, and 3 phases in (HA)_2_(MA)_*n*‑1_Pb_*n*_I_3*n*+1_.^39^ The synthesis of the higher *n* compounds, particularly *n* = 3, required students to explore extensively with Le Chatelier’s principle in mind. A table of known synthesis conditions to achieve all of these crystal growths in HI is given in the Supporting Information Notes for Instructors.

Students were able to qualitatively observe the differences in perovskite crystal growth by examining the crystals under an optical microscope (Figures 5B and C). Drop cast thin films were made by heating the solutions that already contained perovskites to 60–100 °C (redissolving them, the temperature varies between perovskites); then, approximately 10 μL of solution was deposited onto a glass slide on a hot plate in the fume hood, and the solvent was allowed to evaporate. Even though the colors of the crystals usually match what can be observed in the vial, the morphology of the crystals can be very different under the microscope, depending upon the growth conditions. In addition, drop casting is likely how crystals will first be seen from the DMF solutions (which generally do not show visible perovskite crystal growth by simply cooling in the vials). As seen in Figure 5C, the morphology of the crystals grown in DMF is different from that in acid; acid growth tends to form individual crystals and plates, and the DMF growth tends to form an even and more continuous mass of crystals. This reflects how these two solvents are typically used in research: acidic solutions are most often used to grow perovskite single crystals, and DMF is commonly used to make thin films for device applications. In addition to the microscopic images under white light, fluorescent images can also be taken by shining the drop-cast crystals with a UV flashlight with no white light background. This works particularly well with the *n* = 1 crystals, which exhibit a bright green fluorescence that matches their bandgap (Figure 5C).

### Part 2b: Determining the Concentration of Pb in Perovskite Solution Supernatant

Students could immediately observe differences in perovskite solubility after making their samples. For example, in the series of spacer cations (Choice 1), students observed crystal formation for HA-, PEA-, and 4AMP-based perovskites in HI, but not for BA (Figure 6A, top image) using similar precursor recipes. The solutions made with DMF did not appear to yield perovskite crystals at all (Figure 6A, bottom image). To quantitatively measure the difference in perovskite solubility, students performed the same absorbance measurements developed in Part 1 to determine how much soluble lead was leftover in solution. The results that one student group obtained for the spacer cation series in HI can be seen in Figure 6B. The concentration of Pb left in solution for (BA)_2_PbI_4_ is large compared to those of the other samples and explains the lack of crystal formation in the vial. The corresponding percentage of lead removed from solution due to crystal growth can also be calculated, which showed that the samples with the highest percentage of lead removed from solution were the samples with the most crystal growth. The absorbance spectrum of lead in DMF is shown in . Student groups that worked with high *n* samples could also observe trends in the solubility data because excess amounts of lead are needed to drive the perovskite growth to a higher *n* phase ().

**Figure 6:**
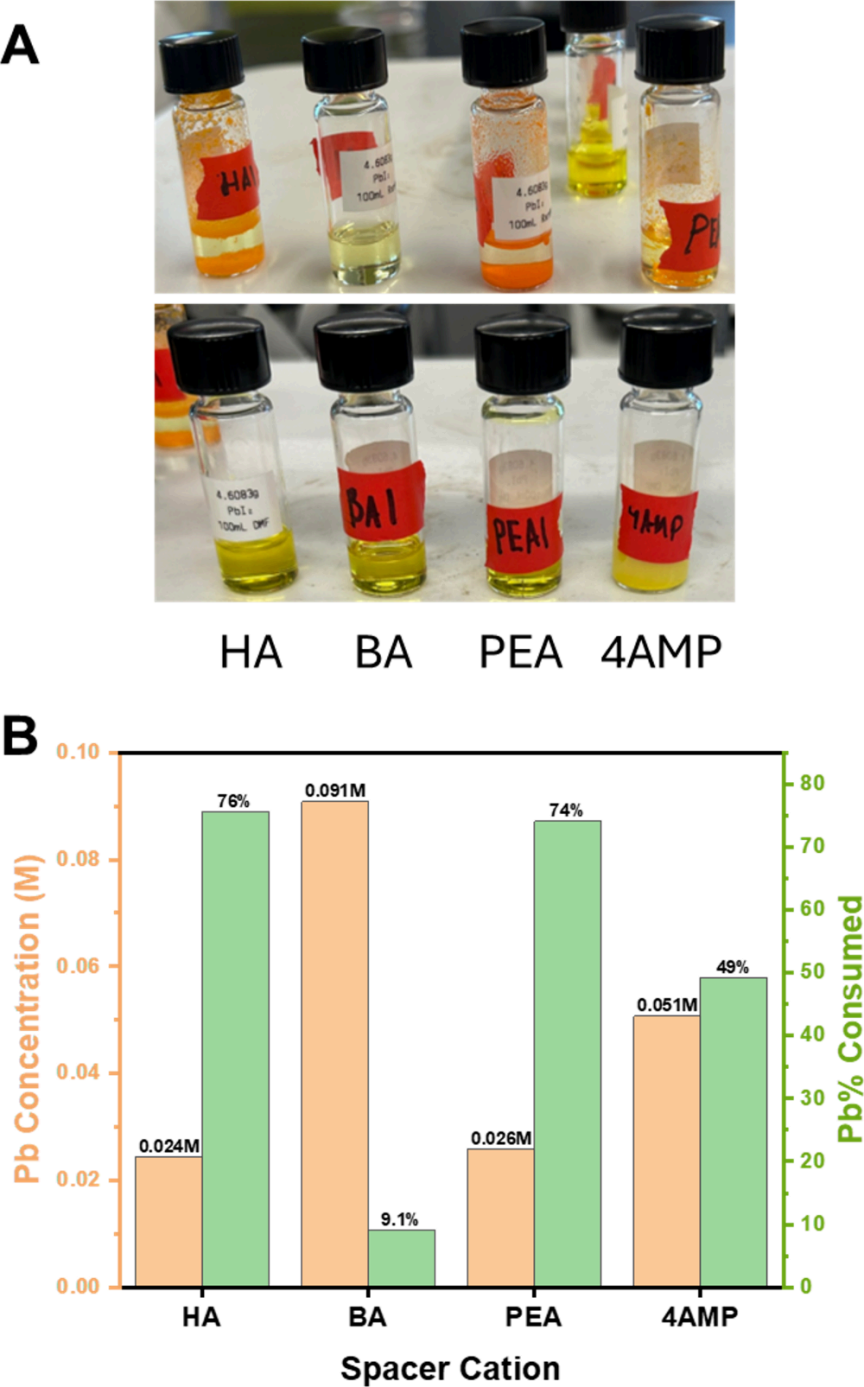
Difference in the perovskite solubility between HI and DMF. A) Images of different (LA)_*m*_PbI_4_ that were all made with 0.1 M PbI_2_ and stoichiometric ratio to LA with top row in HI/H_3_PO_2_ and bottom row in DMF. Note that orange (*n* = 1) perovskites are present in 3 of the 4 top vials (acid solvent), but no solid is present in the bottom vials (DMF). Data was generated by the student group Jaldcorp Solutions Incorporated, which included Annabel C., Laura B., and 2 other students. B) Measured concentrations of Pb remaining in HI solution after growth of perovskite crystals (*n* = 1). Data were collected by the student group DEI (Diffusion, Equilibrium, and Ionization), which included Miguel R-I, Jordan L., Tega I., and another student. Pb concentration in the supernatants from UV–vis absorption (left axis) and corresponding %Pb “consumed” or removed from solution due to crystal growth (right axis). To match the photos in A, if crystals formed, a lower concentration of Pb was found in the solutions (orange bar), resulting in more Pb being consumed (green bar).

### Part 2c: Modification of Spectrometer to Measure Optical Properties of Perovskite Thin Films

Finally, students measured the optical properties that result from the semiconductor nature of the perovskites. Most spectroscopy setups used in undergraduate laboratories are designed for liquid-based samples in cuvettes. However, it is still possible to measure the optical properties of thin films with cuvette-based spectrometers. During the prelab planning phase, students were challenged to figure out how to use the given equipment by sketching out how the spectrometer functioned and debating how a thin-film sample drop cast on glass slides could be inserted into the spectrometers available to them to measure optical properties (Figure 7A). Students needed to think about the optical pathways and how they differ between absorbance and fluorescence measurements in the spectrometer they used. To prepare custom-sized slides, students first measured the cuvette holder and then cut glass slides to fit by scoring them with a diamond-tipped pen and carefully breaking along the score line. The location of the deposited crystals on the glass slide is also important, because they need to be in the optical pathway of the spectrometer. From their microscopy observations in Part 2a, students typically found that samples drop cast from DMF solution produced better crystal coverage with the solvent evaporated more quickly (Figure 7B).

**Figure 7:**
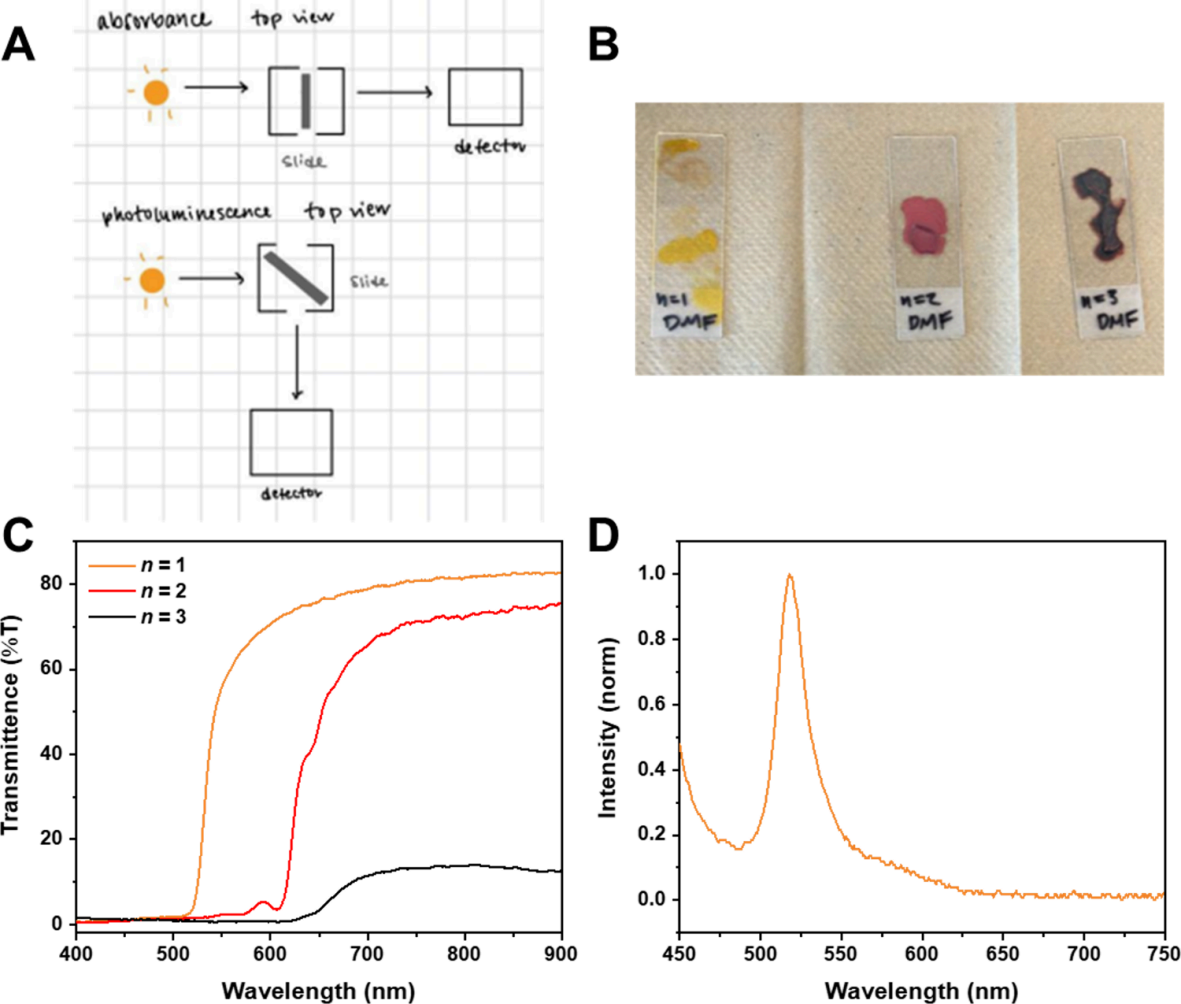
Spectroscopic measurement of perovskite crystals through modifying the cuvette-based spectrometer set up. A) Student sketches from the planning process on how to measure a thin film sample in the provided instrument. Note that a 45° angle is necessary due to the 90° angle between the light source and the detector for photoluminescence. Sketch was generated by the student group LA Lakers, which included Zachary A. and 2 other students. B) Examples of drop cast samples on glass slides. Samples were generated by the student group Triple Threat, which included Ethan P., Lexi R., and Joanna P. C) Percent transmittance measurements in cuvette-based spectrometer for (HA)_2_(MA)_*n*‑1_Pb_*n*_I_3*n*+1_ (*n* = 1–3) samples showing distinct spacing of the transition from transmitting to absorbing, corresponding to the optical bandgaps. Transition should be at approximately 530 nm (*n* = 1), 600 nm (*n* = 2), and 630 nm (*n* = 3).^5^ D) Fluorescence measurement in a cuvette-based spectrometer for (HA)_2_PbI_4_ using a 405 nm LED excitation source showing emission at ∼520 nm, corresponding to the visible green emission under UV light. Fluorescence for *n* = 2 and 3 perovskites was too dim to observe readily with our equipment setup.

Due to an equipment limitation, students measured percent transmittance (%T) on all their perovskite samples instead of absorbance, but this allowed us to introduce the concept of reflectance in spectroscopy. Students have been taught the relation between absorbance and transmittance as *A* = −log­(*T*). However, with solid samples, the light that is delivered to the sample can also be reflected, and therefore, the total light that approaches the sample is a combination of absorbed, transmitted, and reflected. Most cuvette-based spectrometers, including the ones used here, calculate the absorbance through *A* = −log­(*T*) and cannot measure reflectance spectra, so students could only measure the %T of their samples. However, this can still give information very similar to that from absorbance measurements. The %T spectra for (HA)_2_(MA)_*n*‑1_Pb_*n*_I_3*n*+1_ (*n* = 1–3) are shown in Figure 7C. The distinct transitions from a transmitting to absorbing region occur at the optical bandgaps. This measurement allowed students to have quantitative backing for the qualitative color observations they made on crystals of different *n* phases and provided a direct probe of the semiconductor properties of the crystal.

In addition to transmittance, students also attempted to measure the photoluminescence of their samples (Figure 7D). Photoluminescence is a more difficult measurement than transmittance, for several reasons. First, in our set up, the geometry of the glass slide at a 45° angle naturally leads to the reflection of some of the incident light into the detector. This can be fixed either through adding a filter to block the incident light source or through manipulating the sample slide to be slightly off a 45° angle. We encouraged students to use the second method to obtain successful fluorescence spectra, as we did not have the required filters (see Instructor Notes for further details on set up options). Second, the photoluminescence quantum yield – the ratio of the number of photons emitted to number of photons absorbed – varies greatly between perovskites. Some perovskites exhibit bright photoluminescence, while others appear very dim and can be detected only under conditions of high exposure and extended collection times. In general, the *n* = 1 perovskites have a bright green photoluminescence around 520 nm, corresponding to the bandgap, with many students able to identify this relationship between their microscopy, transmittance, and fluorescence data. The photoluminescence of higher *n* perovskites is more difficult to measure, as they generally have much lower quantum yield than the *n* = 1 perovskites.

## Optional Modifications or Additions to Laboratory Experience

The following discussion showcases the wide range of options for running this lab and adapting the content to different courses, equipment availability, and student ability. In addition, while this lab was run as an inquiry-based lab with experiments that instructors believed would work (although not every variation was tested), this lab can easily be modified to fit a CURE, or more research-based experience with true unknowns.

### Perovskite Compositions and Crystal Structures

The experiments listed above can be completed with many variations of perovskite materials. Students can study different variables in perovskite synthesis than those examined here or the focus could be on trying to synthesize new 2D perovskites (i.e., previously unreported).^16–18,36,40,41^ In addition, we note that some of the precursor materials chosen for this experience can be costly. For some guidance on the 2D perovskites that have been reported in the literature and as more options for implementation in this lab experience, see the Supporting Information Notes for Instructors.

### Alternative Solvents

Perovskites can also be grown using several other organic solvents, including dimethyl sulfoxide (DMSO), acetonitrile (ACN), and isopropyl alcohol (IPA). In addition, solvents can be mixed to create unique morphologies and crystal growth outcomes. An interesting version of this lab would focus on the differences in solubility and crystal morphology from solvent choice.

### Powder X-ray Diffraction (PXRD)

2D perovskites are very suitable samples for powder X-ray diffraction (PXRD), as they are highly crystalline and diffract well. Due to their layered structures, distinct, repeating peaks can be seen in the PXRD patterns (), which change with the 2D perovskite layer distance. PXRD is one of the primary ways to confirm the *n* phase of a synthesized 2D perovskite in the research field and is considered to be more accurate than spectroscopic methods. If a PXRD diffractometer is accessible, inorganic chemistry courses could benefit from this addition to the lab.

### ICP­(-MS or -OES)

While the UV–vis absorbance method for determination of lead concentration in solution is a good approximation, ICP measurements of lead concentrations can be more robust. A sample calibration curve for PbI_2_ standards collected by ICP-MS can be found in . This measurement could be done instead of the UV–vis measurements or in parallel, so students could compare the reliability and accuracy of the two methods.

## Student Outcomes

Student success in the stated LOs was assessed through the instructor evaluation of their submitted assignments (including discussion preparation activities and a midpoint check) and a final oral presentation. Each LO was broken down into subsections to fully capture student ability and success (Figure 8A and ). Overall students showed high success rates in LOs that involved synthesis and application of measurements (e.g., LO 2 and LO 4a-c). Students were less successful in connecting experimental results to conceptual content, especially when experimental results did not match expected outcomes (e.g., LO 3c and LO 4d). Some misconceptions remained, which is not unusual in lab settings where students are proposing experimental questions, designing experiments, and analyzing their results in the absence of a known answer. For example, students would sometimes equate environmental stability to how soluble a perovskite crystal was (i.e., thinking degradation = solubility). Or, despite discussing how dissolved PbI_2_ forms a series of lead-iodide complexes (LO 1a), students consistently described the species measured by absorbance as Pb^2+^ (LO 1b). Grade analysis of student presentation results of this lab versus a similar project in the same course showed no statistically significant difference in the final presentation scores (LO 5).

**Figure 8:**
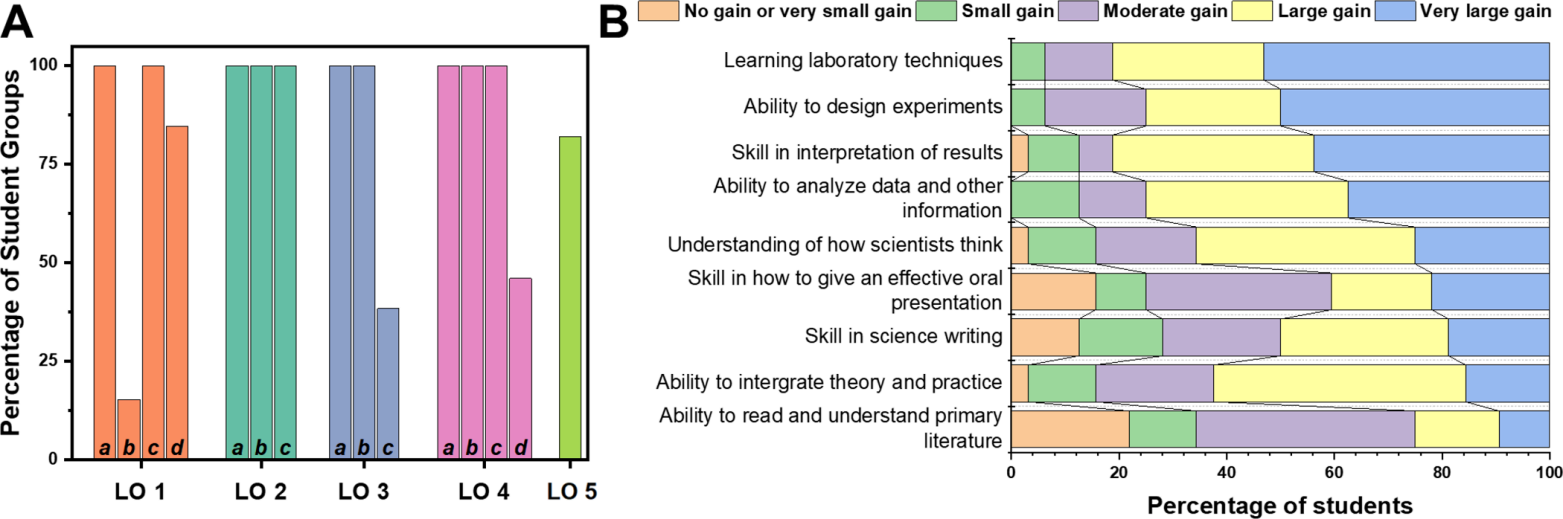
A) Percentage of student groups (*n* = 13) who fulfilled Learning Objectives (LO), broken down by . B) Student (*n* = 32) self-reported skill gains. Survey questions adapted from Lopatto et al.^42^ Skills are arranged from top to bottom from the highest response of “Very large gain” to lowest.

Overall, these results validate that the project lab design was successful in exposing and supporting students to complete the intended learning objectives. Since many of these were only obtained via assessment of submitted documents, many of the students may have met these learning objectives but may not have submitted evidence to that effect (e.g., LOs 1b and 3c). In Table S2, the subaims completion metrics highlight “attempts” (or similar language) as success (e.g., LO 2b, 3b, and 4a). Thus, the previous learning objectives would likely need more direct measures to confirm achievement, but, due to being an inquiry-based experiment, the learning outcomes (and outlined sub aims) were met if students attempted to convey them in their assessments.

In addition to instructor evaluation, students self-reported skill gains through a postsemester survey (*n* = 32 responses on postsurvey out of 40 student class) adapted from Lopatto et al.^42^ where students were asked to rate a list of scientific laboratory skills with a Likert scale to express whether they felt that this lab experience helped to increase their skill in the area. The survey shows that students reported feeling that they had made large learning gains in a variety of skills and abilities from participation in this project lab (Figure 8B). In particular, students reported high gains in practical lab design, execution, and data analysis. Middling areas center on the reporting of results through oral presentations or writing. The lowest reported gain is for reading and understanding the primary scientific literature. While discussion activities and lab manuals pulled figures from publications and students were given reference lists of papers for additional information on perovskite research, there was no scaffolding for reading full journal articles built into the course materials, so this is not a surprising result.^43,44^

Overall feedback on the lab was positive, with students expressing that even if they were not planning on pursuing careers in material chemistry or science, they still found the lab interesting and rewarding, such as “I have pretty much already decided my career path, and the project lab isn’t really related to this path, but I did find it pretty interesting”. Students who were interested in material science expressed extremely positive views on the lab, like “I was already interested in materials chemistry, so learning about perovskites was fun”. Some challenges were expressed in the high workload and stress, although this feedback was generally given in context with the concurrent lecture portion of the course, and efforts were made to adjust the schedule and accommodate concerns. This was the first time most students have participated in a research-like experience, and therefore the higher mental workload and working with unknown outcomes was a new experience for many.^45^ However, the positive response indicates to us that continuing this lab into the future will be rewarding for students.

## Conclusion

In summary, this work describes a flexible, inquiry-based chemistry laboratory experience surrounding halide perovskite materials. During the process of successfully synthesizing and characterizing a multitude of perovskite samples, students designed their own experiments to answer questions relating to perovskite synthesis, structure, and properties. Specifically, students are introduced to topics such as crystal structures, complexation, solubility, microscopy, and spectroscopy as they relate to semiconductor materials. While the current design provides considerable scaffolding to support student success, future iterations may reduce guided elements to allow for a more authentic CURE experience with deeper engagement in open-ended, research-like inquiry. Students reported high gains in their ability to design and execute experiments and process experimental data and an overall positive view of the lab experience. The experiments and characterization techniques can be customized to budget and equipment availability, while still maintaining the ability to examine core chemistry principles as applied to cutting-edge materials research. Although this lab took place in an analytical chemistry classroom, the content can be easily adapted for an inorganic or physical chemistry course or for material science and engineering courses. Overall, this experiment offers a method for introducing a current materials science research topic into the undergraduate classroom while teaching the fundamental chemistry principles required to be covered in the course.

## Supplementary Material





















## Notes

Notes for Instructors, including supplemental figures and discussion, chemical and equipment lists, and full procedures (PDF, DOCX)
Lab Manual for Part 1 (PDF, DOCX)
Lab Manual for Part 2 (PDF, DOCX)
Prelab Discussion Activity for Part 1 (PDF, DOCX)
Prelab Discussion Activity for Part 2 (PDF, DOCX)

## References

[1] Fu Y., Zhu H., Chen J., Hautzinger M. P., Zhu X.-Y., Jin S. (2019). Metal Halide
Perovskite Nanostructures for Optoelectronic Applications and the
Study of Physical Properties. Nat. Rev. Mater..

[2] Liu X.-K., Xu W., Bai S., Jin Y., Wang J., Friend R. H., Gao F. (2021). Metal Halide
Perovskites for Light-Emitting Diodes. Nat.
Mater..

[3] Stranks S. D., Snaith H. J. (2015). Metal-Halide Perovskites for Photovoltaic
and Light-Emitting
Devices. Nat. Nanotechnol..

[4] Stoumpos C. C., Cao D. H., Clark D. J., Young J., Rondinelli J. M., Jang J. I., Hupp J. T., Kanatzidis M. G. (2016). Ruddlesden-Popper
Hybrid Lead Iodide Perovskite 2D Homologous Semiconductors. Chem. Mater..

[5] Cao D. H., Stoumpos C. C., Farha O. K., Hupp J. T., Kanatzidis M. G. (2015). 2D Homologous
Perovskites as Light-Absorbing Materials for Solar Cell Applications. J. Am. Chem. Soc..

[6] Shekhirev M., Goza J., Teeter J. D., Lipatov A., Sinitskii A. (2017). Synthesis
of Cesium Lead Halide Perovskite Quantum Dots. J. Chem. Educ..

[7] Cherrette V. L., Hutcherson C. J., Barnett J. L., So M. C. (2018). Fabrication and
Characterization of Perovskite Solar Cells: An Integrated Laboratory
Experience. J. Chem. Educ..

[8] Yang H., Fan W., Hills-Kimball K., Chen O., Wang L.-Q. (2019). Introducing
Manganese-Doped Lead Halide Perovskite Quantum Dots: A Simple Synthesis
Illustrating Optoelectronic Properties of Semiconductors. J. Chem. Educ..

[9] Lisensky G. C., Dauzvardis F., Young M. M. K. (2021). Periodic Properties Illustrated by
CH _3_ NH _3_ Pb­(I _1‑*x*
_ Br _
*x*) 3_ Solid Solution Perovskite
Semiconductors. J. Chem. Educ..

[10] Qiu S., Zhang D., Yeddu V., Cordoba C., Blackburn A. M., Iosub V., Saidaminov M. I. (2023). The Importance of Synthesis Conditions:
Structure-Processing-Property Relationships. J. Chem. Educ..

[11] Wallace M. K., Cooke J. (2025). Synthesis of Luminescent Mn2+ and
Sb3+ Doped Chloride Double Perovskites:
An Adaptable, Inquiry-Based Experiment for Short Laboratory Sessions. J. Chem. Educ..

[12] Mejía
Vázquez M. C., Bernal W., Gómez Téllez A. C., Camacho Cáceres J., Montoya Montoya D. M., Pacio M., Hu H. (2024). Synthesis, Fabrication, and Characterization
of MAPbBr3 Quantum Dots for LED Applications: An Easy Laboratory Practice. J. Chem. Educ..

[13] Triggs C. T., Ross R. D., Mihalyi-Koch W., Clewett C. F. M., Sanders K. M., Guzei I. A., Jin S. (2024). Spacer Cation
Design Motifs for Enhanced
Air Stability in Lead-Free 2D Tin Halide Perovskites. ACS Energy Lett..

[14] Hossain T., Atapattu H. R., Pruett H., Rahman M. T., Pedersen K. R., Huckaba A. J., Parkin S. R., Graham K. R. (2024). Effects of A-Site
Cation Structure on the Stability of 2D Tin Halide Perovskites. Chem. Mater..

[15] Grancini G., Roldán-Carmona C., Zimmermann I., Mosconi E., Lee X., Martineau D., Narbey S., Oswald F., De Angelis F., Graetzel M., Nazeeruddin M. K. (2017). One-Year Stable Perovskite Solar
Cells by 2D/3D Interface Engineering. Nat. Commun..

[16] Li X., Hoffman J. M., Kanatzidis M. G. (2021). The 2D
Halide Perovskite Rulebook:
How the Spacer Influences Everything from the Structure to Optoelectronic
Device Efficiency. Chem. Rev..

[17] Saparov B., Mitzi D. B. (2016). Organic-Inorganic Perovskites: Structural Versatility
for Functional Materials Design. Chem. Rev..

[18] Smith M. D., Crace E. J., Jaffe A., Karunadasa H. I. (2018). The Diversity
of Layered Halide Perovskites. Annu. Rev. Mater.
Res..

[19] Mao L., Stoumpos C. C., Kanatzidis M. G. (2019). Two-Dimensional
Hybrid Halide Perovskites:
Principles and Promises. J. Am. Chem. Soc..

[20] Smith M. D., Connor B. A., Karunadasa H. I. (2019). Tuning the Luminescence of Layered
Halide Perovskites. Chem. Rev..

[21] Auchincloss L. C., Laursen S. L., Branchaw J. L., Eagan K., Graham M., Hanauer D. I., Lawrie G., McLinn C. M., Pelaez N., Rowland S., Towns M., Trautmann N. M., Varma-Nelson P., Weston T. J., Dolan E. L. (2014). Assessment of Course-Based
Undergraduate Research Experiences: A Meeting Report. LSE.

[22] Buchanan A. J., Fisher G. R. (2022). Current Status and Implementation of Science Practices
in Course-Based Undergraduate Research Experiences (CUREs): A Systematic
Literature Review. LSE.

[23] Watts F. M., Rodriguez J.-M. G. (2023). A Review
of Course-Based Undergraduate Research Experiences
in Chemistry. J. Chem. Educ..

[24] Jegstad K. M. (2024). Inquiry-Based
Chemistry Education: A Systematic Review. Studies
in Science Education.

[25] Pedaste M., Mäeots M., Siiman L. A., de Jong T., van Riesen S. A. N., Kamp E. T., Manoli C. C., Zacharia Z. C., Tsourlidaki E. (2015). Phases of
Inquiry-Based Learning: Definitions and the Inquiry Cycle. Educational Research Review.

[26] Eberlein T., Kampmeier J., Minderhout V., Moog R. S., Platt T., Varma-Nelson P., White H. B. (2008). Pedagogies of Engagement in Science:
A Comparison of PBL, POGIL, and PLTL. Biochem
Molecular Bio Educ.

[27] Lee, J. S. ; Blackwell, S. ; Drake, J. ; Moran, K. A. Taking a Leap of Faith: Redefining Teaching and Learning in Higher Education Through Project-Based Learning. Interdisciplinary Journal of Problem-Based Learning 2014, 8 (2), 10.7771/1541-5015.1426.

[28] Schwarz G. (2025). Literature
Survey on Quantitative Chemical Analyses Experiments for Students:
Exploring Choices. J. Chem. Educ..

[29] Buck L. B., Bretz S., Towns M. (2008). Characterizing the Level of Inquiry
in the Undergraduate Laboratory. Journal of
College Science Teaching.

[30] Van
Wyk A. L., Frederick K. A., Lieberman M., Cole R. S. (2025). Increasing Authenticity of the Laboratory through the
MICRO Project: Analysis of Analytical Chemistry Laboratory Experiments
for Their Level of Inquiry. J. Chem. Educ..

[31] Kovarik M. L., Galarreta B. C., Mahon P. J., McCurry D. A., Gerdon A. E., Collier S. M., Squires M. E. (2022). Survey of the Undergraduate Analytical
Chemistry Curriculum. J. Chem. Educ..

[32] Horváth O., Mikó I. (1998). Spectra, Equilibrium and Photoredox Chemistry of Tri-
and Tetraiodoplumbate­(II) Complexes in Acetonitrile. J. Photochem. Photobiol., A.

[33] Stamplecoskie K. G., Manser J. S., Kamat P. V. (2015). Dual Nature
of the Excited State
in Organic-Inorganic Lead Halide Perovskites. Energy Environ. Sci..

[34] Harris, D. C. ; Lucy, C. A. Quantitative Chemical Analysis, 10th ed.; Macmillan Learning: New York, 2020.

[35] Pan D., Fu Y., Spitha N., Zhao Y., Roy C. R., Morrow D. J., Kohler D. D., Wright J. C., Jin S. (2021). Deterministic Fabrication
of Arbitrary Vertical Heterostructures of Two-Dimensional Ruddlesden-Popper
Halide Perovskites. Nat. Nanotechnol..

[36] Mihalyi-Koch W., Folpini G., Roy C. R., Kaiser W., Wu C.-S., Sanders K. M., Guzei I. A., Wright J. C., De Angelis F., Cortecchia D., Petrozza A., Jin S. (2023). Tuning Structure and
Excitonic Properties of 2D Ruddlesden-Popper Germanium, Tin, and Lead
Iodide Perovskites via Interplay between Cations. J. Am. Chem. Soc..

[37] Fu Y., Hautzinger M. P., Luo Z., Wang F., Pan D., Aristov M. M., Guzei I. A., Pan A., Zhu X., Jin S. (2019). Incorporating Large A Cations into
Lead Iodide Perovskite Cages:
Relaxed Goldschmidt Tolerance Factor and Impact on Exciton-Phonon
Interaction. ACS Cent. Sci..

[38] Hautzinger M. P., Mihalyi-Koch W., Jin S. (2024). A-Site Cation Chemistry in Halide
Perovskites. Chem. Mater..

[39] Spanopoulos I., Hadar I., Ke W., Tu Q., Chen M., Tsai H., He Y., Shekhawat G., Dravid V. P., Wasielewski M. R., Mohite A. D., Stoumpos C. C., Kanatzidis M. G. (2019). Uniaxial Expansion of the 2D Ruddlesden-Popper Perovskite
Family for Improved Environmental Stability. J. Am. Chem. Soc..

[40] Forlano K. M., Roy C. R., Mihalyi-Koch W., Hossain T., Sanders K., Guzei I., Graham K. R., Wright J. C., Jin S. (2023). High Layer
Number (*n* = 1–6) 2D Ruddlesden-Popper Lead
Bromide Perovskites: Nanosheets, Crystal Structure, and Optoelectronic
Properties. ACS Materials Lett..

[41] Guo S., Mihalyi-Koch W., Mao Y., Li X., Bu K., Hong H., Hautzinger M. P., Luo H., Wang D., Gu J., Zhang Y., Zhang D., Hu Q., Ding Y., Yang W., Fu Y., Jin S., Lü X. (2024). Exciton Engineering
of 2D Ruddlesden-Popper Perovskites by Synergistically Tuning the
Intra and Interlayer Structures. Nat. Commun..

[42] Lopatto D. (2004). Survey of
Undergraduate Research Experiences (SURE): First Findings. CBE.

[43] Kovarik M. L. (2016). Use of
Primary Literature in the Undergraduate Analytical Class. Anal Bioanal Chem..

[44] Hunter R. A., Kovarik M. L. (2022). Leveraging the Analytical
Chemistry Primary Literature
for Authentic, Integrated Content Knowledge and Process Skill Development. J. Chem. Educ..

[45] Buchberger A. R., Mill J. (2025). The Effects of Delaying
Physical Laboratory Experiences on Student
Self-Efficacy. J. Chem. Educ..

