# Supplementary material for: Shining Light on Halide Perovskites: Teaching Analytical Chemistry Using Flexible, Inquiry-Based Experiments
Source: J Chem Educ. 2026 Feb 19;103(3):1480–90. doi: 10.1021/acs.jchemed.5c00906 (PMC12980825; doi:10.1021/acs.jchemed.5c00906)
Supplement: Supplementary file 2 [file ed5c00906_si_003.pdf]

Shining Light on Halide Perovskites: Teaching Analytical Chemistry Using Flexible, Inquiry-Based Experiments

Kristel M. Forlano, Eliana Bernat, Pamela Doolittle, Dominic Colosi, Song Jin*, Amanda Rae Buchberger*

Department of Chemistry, University of Wisconsin – Madison, Madison, WI, 53706, United States

*Email:
amanda.buchberger@wisc.edu
jin@chem.wisc.edu

Notes for Instructors

Table of Contents:

1. **Chemical and equipment list**
   Table of chemicals used S2
   Equipment used S3
2. **Additional background on halide perovskites** S4
3. **Experimental notes for instructors** S7
   Part 1: Solubility of PbI_2 S7
   Part 2: Exploration of 2D perovskites S9
      Part 2a: Synthesis and Microscopy S10
      Part 2b: Perovskite solubility S14
      Part 2c: Measuring optical properties S16
   Additional instructor notes S17
4. **Notes on optional modification to lab** S18
5. **Notes on Learning Objectives and Outcomes** S22
6. **Additional video resources for students and instructors** S24
7. **Full course schedule** S25

1. Chemical and equipment list

Chemicals list

These amounts were calculated for 13 student groups from the suggested volumes and concentrations given in the lab manuals. Excess was purchased for student error and exploration.

Table S1. Chemicals used in reported lab experiments.

Chemical Name	CAS Number	Amount Purchased	Supplier	Cost*
Hydroiodic acid (57 Wt.%)	10034-85-2	8 x 250 mL	Sigma-Aldrich	$241.88 each
Hypophosphorous acid	6303-21-5	2 kg	VWR, Beantown Chemical	$258.38
Lead (II) iodide (99%)	10101-63-0	250 g	ChemImpex	$140.01
n-Hexylammonium iodide	54285-91-5	50 g	Greatcell solar materials	$392.00
4-(Aminomethyl)piperidine	7144-05-0	25 g	TCI America	$87.79
n-Butylammonium iodide	36945-08-1	10 g	Greatcell solar materials	$71.96
Phenethylammonium iodide	151059-43-7	10 g	Greatcell solar materials	$79.43
Methylammonium iodide	4965-49-2	50 g	Greatcell solar materials	$124.90
Formamidinium iodide	879643-71-7	10 g	Greatcell solar materials	$76.69
Guanidinium iodide	19227-70-4	10 g	Greatcell solar materials	$48.85

*Cost is from time of purchase in January and February 2025

- Many of the spacer cations and A-cations are hydroscopic so should be capped immediately after use

Equipment used:

- 2 dram vials (~7 mL)
- Wiretrols (5/10, 25, 50/100 μL)
- Hot plates
- Glass slides and glass scoring tools
- UV flashlight, 5 W 395 nm
- Quartz cuvettes that can be used in the UV range (at least 330 nm)
- Red Tide UV-Vis spectrometers (measures 200-850 nm) that connect to Vernier Technology (*i.e.*, LabQuests)
  - For measuring solutions in Part 1 & 2
- Agilent, Cary 5000 UV–Vis–NIR spectrophotometer
  - For instructor and curriculum developer solution measurements
- American Optical Model Fifty binocular microscopes with attached USB Swift EC Series Microscope Digital camera (model EC5R)
  - Swift Imaging 3.0 software to capture images and video
- Jasco Model V-570 UV/VIS/NIR spectrometer
  - For measuring solutions in Part 2 only
- Vernier SpectroVis® Plus Spectrophotometer with attached LabQuests (405 nm and 525 nm LED excitations sources)
  - For measurement of thin films in Part 2

2. Background on Semiconductor Properties of 2D Halide Perovskites

A semiconductor can be broadly defined based on the energy band structure of a material, specifically the relation between the valence band and the conduction band (Figure S1a). In the simplest representation, the valence band, which is the lower energy band, contains the electrons in a material. In order for a material to conduct electricity, electrons must be in the conduction band, which has a higher energy than the valence band. In a metal, these bands overlap each other, so electrons can freely move into the conduction band and travel around the material. In a semiconductor, the bands have some energy difference between them called a "bandgap.", If the energy of that difference is applied to that material, an electron can be promoted from the valence band to the conduction band, therefore allowing the material to conduct electricity under certain conditions, or semi-conductor. In an insulator, these bands are too far apart in energy for the electron to travel between. The properties of semiconductors make them suitable for many optoelectronic device applications. For example, when an electron is promoted by light excitation to the conduction band, it leaves behind a positive charge, aptly named a "hole." Devices can be designed so that electrons and holes flow in opposite directions across the device architecture, generating a current. This is how solar cells operate.[1] On the other hand, the electron and hole are attracted together as a quasi-particle called an "exciton", due to the opposite charges of each particle (Coulombic attraction).[2] An electron can lose the energy that was used to promote it to the conduction band and recombine with the hole. The energy lost is generally emitted in the form of light, or photoluminescence. This is the mechanism behind light emitting diodes (LEDs).[3]

In semiconductors, the bandgap is determined by the atoms, their crystal structures, and atomic orbital overlap that make up the material. For perovskites, the orbitals that make up the top of the valence band and the bottom of the conduction band come from the B-X inorganic octahedra, so therefore changing the B- or X-site changes the bandgap. However, in nanomaterials, there is one other influence factor: the physical size.[4] As shown in Figure S1B, the exciton discussed earlier occupies a certain physical space in the material. However, when the material itself is smaller than the space that the excitons occupy, the material is said to be "quantum-confined". This causes many interesting phenomena in semiconductors, including changes to the bandgap and higher exciton binding energy.[5,6] Quantum confined materials are of great interest to the research community due to their unique properties and applications, and one form, quantum dots (semiconductor nanoparticles), was recently recognized with the 2023 Nobel Prize in chemistry (https://www.nobelprize.org/prizes/chemistry/2023/press-release/). 3D perovskite materials do not exhibit quantum confinement (unless as very small perovskite quantum dots). However, the 2D perovskites do. The one-to-few layers of inorganic octahedra that

form the layers of the 2D perovskite structure are smaller than the exciton radius, and therefore the material exhibits quantum confinement. Between altering the B-site, X-site, and *n* number, the bandgap of 2D perovskites is altered.[7] This can be clearly illustrated by the photoluminescence response of various 2D perovskites with X = I or Br and different *n* numbers (Figure S1c).[8,9]

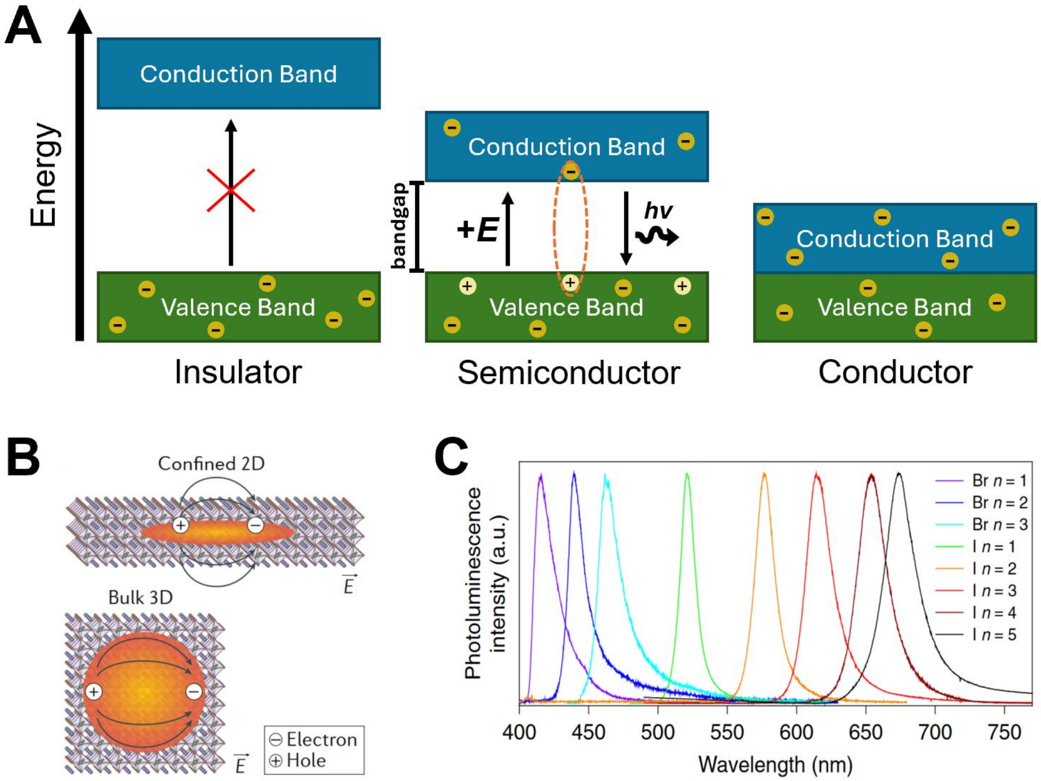

Figure S1. Properties of perovskite semiconductors. A) Schematic diagrams showing differences in band energy structure between insulators, semiconductors, and conductors. Exciton quasiparticle in semiconductor indicated by dashed orange line. B) Diagram of exciton quantum confinement in 2D perovskite materials. (Reprinted with permission from Fu, Y., Zhu, H., Chen, J. *et al.* Metal halide perovskite nanostructures for optoelectronic applications and the study of physical properties. *Nat Rev Mater* **4**, 169–188 (2019). Copyright 2019 Nature). C) Photoluminescence spectra of various 2D RP perovskites showing PL dependence on halide and *n* number composition. (Reprinted with permission from Pan, D., Fu, Y., Spitha, N. *et al.* Deterministic fabrication of arbitrary vertical heterostructures of two-dimensional Ruddlesden–Popper halide perovskites. *Nat. Nanotechnol.* **16**, 159–165 (2021). Copyright 2021 Nature.)

References

(1) Green, M. A.; Ho-Baillie, A.; Snaith, H. J. The Emergence of Perovskite Solar Cells. *Nature Photon* **2014**, *8*, 506–514.

(2) Marongiu, D.; Saba, M.; Quochi, F.; Mura, A.; Bongiovanni, G. The Role of Excitons in 3D and 2D Lead Halide Perovskites. *J. Mater. Chem. C* **2019**, *7*, 12006–12018.

(3) Veldhuis, S. A.; Boix, P. P.; Yantara, N.; Li, M.; Sum, T. C.; Mathews, N.; Mhaisalkar, S. G. Perovskite Materials for Light-Emitting Diodes and Lasers. *Advanced Materials* **2016**, *28*, 6804–6834.

(4) Fu, Y.; Zhu, H.; Chen, J.; Hautzinger, M. P.; Zhu, X.-Y.; Jin, S. Metal Halide Perovskite Nanostructures for Optoelectronic Applications and the Study of Physical Properties. *Nat Rev Mater* **2019**, *4*, 169–188.

(5) Leng, K.; Fu, W.; Liu, Y.; Chhowalla, M.; Loh, K. P. From Bulk to Molecularly Thin Hybrid Perovskites. *Nat Rev Mater* **2020**, *5*, 482–500.

(6) Mauck, C. M.; Tisdale, W. A. Excitons in 2D Organic–Inorganic Halide Perovskites. *Trends in Chemistry* **2019**, *1*, 380–393.

(7) Blancon, J.-C.; Even, J.; Stoumpos, Costas. C.; Kanatzidis, Mercouri. G.; Mohite, A. D. Semiconductor Physics of Organic–Inorganic 2D Halide Perovskites. *Nat. Nanotechnol.* **2020**, *15*, 969–985.

(8) Pan, D.; Fu, Y.; Spitha, N.; Zhao, Y.; Roy, C. R.; Morrow, D. J.; Kohler, D. D.; Wright, J. C.; Jin, S. Deterministic Fabrication of Arbitrary Vertical Heterostructures of Two-Dimensional Ruddlesden–Popper Halide Perovskites. *Nat. Nanotechnol.* **2021**, *16*, 159–165.

(9) Smith, M. D.; Connor, B. A.; Karunadasa, H. I. Tuning the Luminescence of Layered Halide Perovskites. *Chem. Rev.* **2019**, *119*, 3104–3139.

3. Experimental Notes for Instructors

The Part 1 Discussion Activity thoroughly goes over the conceptual content for Part 1. Students were expected to generate their own procedures based on the guiding questions presented in the lab manuals.

Part 1: Solubility of PbI_2 in water vs acid

Any reference to HI is a 1:1 v/v mix of 57% HI and H_3PO_2.

Prelab instructor-made solutions:

- 0.1 M PbI_2 stock solution in 1:1 v/v of HI:H_3PO_2
- Saturated PbI_2 solutions on a 1 mL scale in:
  - 1:0 HI (~600 mg PbI_2 required)
  - 75:25 HI:H_2O (~400 mg PbI_2 required)
  - 50:50 HI:H_2O (~200 mg PbI_2 required)
  - 25:75 HI:H_2O (~75 mg PbI_2 required)

Each student group was given to start with:

- 40 mL of HI
- 3 mL of 0.1 M PbI_2 stock solution
- 1 vial of HI saturated with PbI_2
- 1 vial of HI:H_2O saturated with PbI_2
  - A student group only needed to work with one ratio, then shared their data with the rest of the class.

Additional notes:

- The calibration curve made will likely have a y-intercept that is well below zero (see Figure 4B). We believe that this is due to matrix effects in the solution, or because this is a proxy measurement for Pb concentration. The Part 1 discussion activity discusses how this absorbance peak only relates to the $[PbI_3]^-$ concentration. The other lead iodide complexes are not taken into account with this method.
- Saturated PbI_2 solutions were made to have significant solid leftover after stirring and sitting overnight.
- Students were given the actual concentration of the 0.1 M PbI_2 stock solution, as measured out by the instructional staff, to make an accurate calibration curve as well as the amount of PbI_2 in their saturated solutions to see how different the amount of PbI_2 needed to saturate the solution was.

- Most student groups were able to do this within the given lab time. The most difficult part of this experiment is to get the concentration of the saturated solution into the range of the calibration curve. This could take many attempts, and consume a lot of solvent, if students are not thoughtful.
- Collected class data is seen in Figure 4C. We believe that the variability of the calculated solubility between student groups is due to the solid PbI_2 that should have been filtered out while taking aliquots of supernatant or poor technique when using the wiretrols. These are changes that we plan to make when we run this lab again.
    - Students performed statistical analysis on the whole class data. There were 4-5 student groups that looked at each $HI:H_2O$ ratio. With only a few data points for each sample and a wide spread in measured concentration, students were not able to discard any data points through a Grubb's test.

Part 2: Exploration of 2D perovskites

There were three facets to the Part 2 experiments: Synthesis and microscopy, solution spectroscopy, and solid-state spectroscopy. The Part 2 Discussion Activity is critical for introducing students to the perovskite crystal structure and semiconductor properties.

All student groups synthesize $(HA)_2PbI_4$ as a control as this crystal is easy to grow and can be part of the comparison between different perovskites for each of the variable choices described below. A generic procedure for perovskite synthesis is given in the Part 2 Lab Manual. In general, students will see crystals form in the vials in HI and not in DMF (these are seen only when drop casting).

Prelab instructor-made solutions:

- 1 mL aliquots 0.1 M, 0.3 M, and 0.5 PbI_2 in 1:1 v/v of HI:H_3PO_2 in 2 dram vials
- 1 mL aliquots 0.1 M, 0.3 M, and 0.5 PbI_2 in DMF in 2 dram vials

Students checked these vials out of the stockroom as needed. They could request that other concentrations were made to help them achieve certain crystal growths on a case-by-case basis. Students made the perovskite crystals by adding the remaining perovskite precursors directly to these PbI_2 solution vials.

For control $(HA)_2PbI_4$ sample, students were given:

- 1 g HAI

Part 2a: Synthesis and microscopy of perovskite crystals

Students were able to pick a design variable in perovskites to explore. There were a set number of spots for each option so that the distribution would be even and for estimating material purchasing amounts. Known synthesis conditions for all perovskite crystals are given in Table S2.

Table S2. Precursor concentrations to grow 2D perovskite crystals in HI.

Crystal	LA (M)	A (M)	PbI_2 (M)
$(HA)_2PbI_4$	0.2	-	0.1
$(BA)_2PbI_4$	0.4	-	0.2
$(PEA)_2PbI_4$	0.2	-	0.1
$(4AMP)PbI_4$	0.1	-	0.1
$(HA)_2(MA)Pb_2I_7$	0.15	0.25	0.5
$(HA)_2(FA)Pb_2I_7$	0.05	0.125	0.45
$(HA)_2(GA)Pb_2I_7$	0.05	0.7	0.5
$(HA)_2(MA)_2Pb_3I_{10}$	0.08	0.42	0.5

Choice 1 variable: Spacer cation

Students were given:

- 1 g of BAI and PEAI
- 0.5 mL of 4AMP

Notes for this option:

- Because these are all n = 1 crystals, they will likely be easy to grow.
- Students will notice differences in solubility that they can link back to the structure of the spacer cation.
- At 0.1 M concentration, $(BA)_2PbI_4$ is unlikely to grow in HI because it is a very soluble crystal (see Figure 6 in main text). Increasing the concentration will make crystals grow.
- There are two forms of 2D perovskite: Ruddlesden-Popper (RP), which uses monoammonium spacer cation and forms a bilayer of cations, and Dion-Jacobson (DJ), which uses a diammonium spacer cation and forms a monolayer of cations (Figure S2). 4AMP is the only DJ spacer cation used in this activity.

- We purchased 4AMP in the amine form (instead of the ammonium iodide salt) due to cost and availability. This was also the only cation that was a liquid.
    - Students needed to consider density when calculating amount to add to their synthesis.
    - In HI, 4AMP is protonated and therefore can form the perovskite. This is not the case in DMF and therefore crystals will not form. Students did notice this difference, and many were able to correctly identify the cause.
        - The 4AMPI salt can be made by adding an excess of 4AMP to HI. This is an easy process, but exothermic (combining acid and base) so safety precautions should be taken.

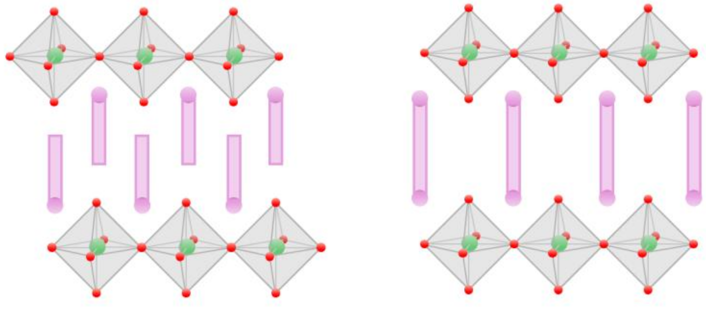

Figure S2. Structure difference between Ruddlesden-Popper (RP) and Dion-Jacobson (DJ) perovskites.

Choice 2 variable: A-site cation

Students were given:

- 3 g of HAI (in addition to control sample amount)
- 1.5 g of MAI, FAI, and GAI

Notes for this option:

- Students may need to tune the synthesis for these $n = 2$ crystals. Stoichiometric amounts are not guaranteed to work (see Figure S3A for example of mixed synthesis)
- $(HA)_2(GA)Pb_2I_7$ is the most difficult crystal to form due to the large size of the GA cation. High ratios of GA and Pb to HA are needed.
    - Most likely the $n = 1$ $(HA)_2PbI_4$ will continue to form until reaction is driven far enough

Choice 3 variable: n number

Students were given:

- 2 g of HAI (in addition to control sample amount)
- 2g of MAI

Notes for this option:

- The *n* = 3 crystal is likely one of the most difficult to synthesize in these experiments. Students may need to make several attempts or request higher PbI_2 stock solutions (see Figure S3A for example of mixed *n* synthesis).
  - This is also the most likely synthesis where the solution may become overconcentrated. Students still need access to the mother liquor (*i.e.* the solvent that is used to grow crystals) to complete the spectroscopy experiments. While it is tempting for students to try to drive this reaction by continuing to add more MA and Pb, decreasing the spacer cation also accomplishes the same thing and can decrease the overall concentration of the solution.
  - This synthesis may also see white crystals crash out of solution. These are the precursor salts (HAI, MAI) and indicate a saturation and/or insolubility of that precursor (Figure S3B)

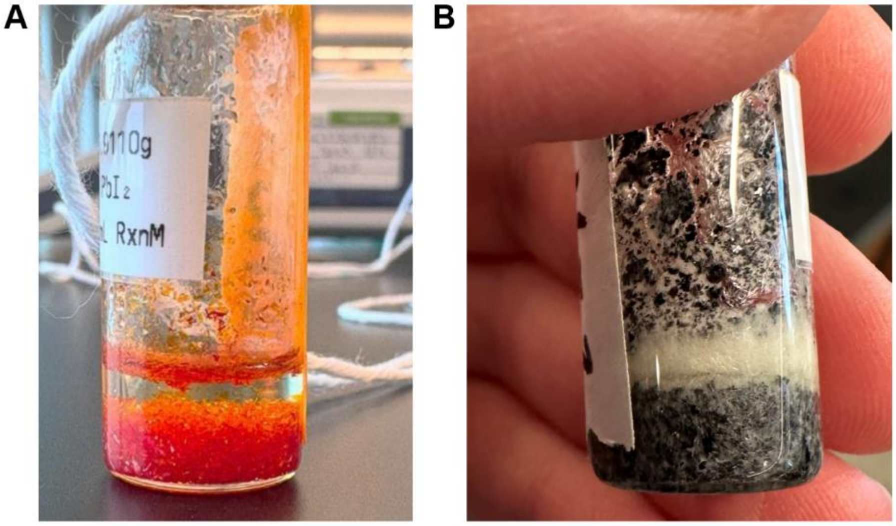

Figure S3. Images of student perovskite syntheses showing possible outcomes. A) Mixed *n* phase with both orange (*n* = 1) and red (*n* = 2) crystals and B) white precursor salt crystals growing in black (*n* = 3) perovskite solution.

Notes on Microscopy:

- It's good practice for students to get used to adding scale bars to their images. Most microscope camera software has this setting available. Otherwise, software like ImageJ can be used if you manually calculate a pixel-to-distance ratio.
- One microscope set up was placed in a dark room to help students acquire better fluorescence images using a UV flashlight
- While fluorescence images could only be captured for the $n = 1$ perovskites with our set up, if a more advanced microscope or spectrometer is available that allows for long exposure and collection times, the photoluminescence for lead iodide perovskites is expected to be around 570 nm (orange) for $n = 2$ and 620 nm (red) for $n = 3$.

Part 2b: Determining the concentration of Pb in perovskite solution supernatant

NOTE: We had instructor-made calibration solutions of PbI_2 in HI available for all students during this part of the lab.

This set of experiments functions similarly to Part 1 with the addition of using DMF as a solvent. A full spectrum of PbI_2 in DMF is shown in Figure S5. Similar to HI, the peak at approximately 360 nm can be attributed to the $[PbI_3]^-$ complex. Students should always be taking full spectrum measurements of their samples to notice the slight peak shift between the HI and DMF solutions. We did not have students create a new calibration curve for DMF samples, so this approximation and source of error should be noted for both instructors and students. In addition, at some concentrations of PbI_2 in DMF other peaks can be observed in the absorbance spectrum that can be attributed to the other lead iodide complexes (See Part 1 Discussion Activity), which would naturally lead to an underestimate of the soluble lead concentration. The discussion should be had with students about method accuracy and validation.

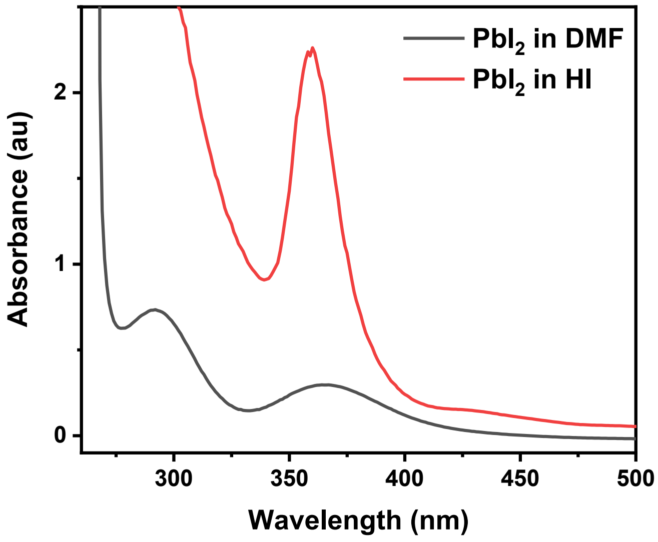

Figure S4. Absorbance spectra of PbI_2 in DMF and HI.

The different variable choices will notice different trends in their solubility data (Figure 6 in main text). Choices 2 and 3, where higher *n* are needed, will notice that the increased amount of Pb needed for their syntheses is reflected in the absorbance data (Figure S5).

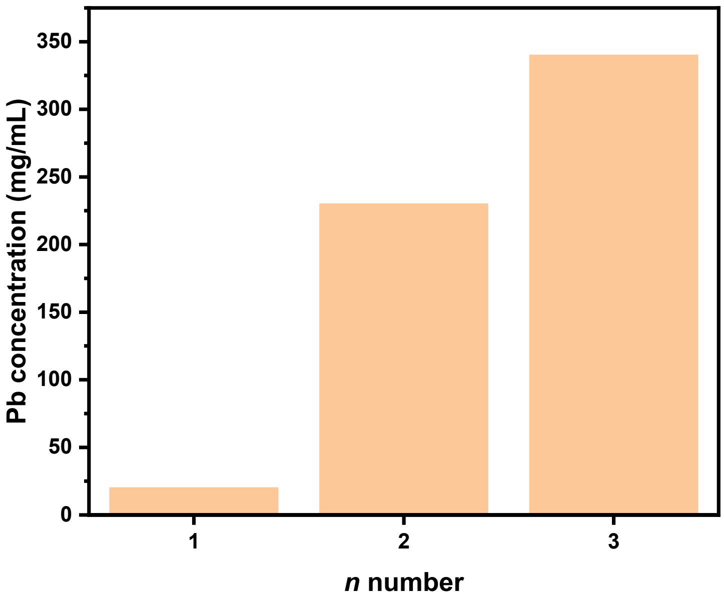

Figure S5. Change in Pb concentration in different *n* samples of $(HA)_2(MA)_{n-1}Pb_nI_{3n+1}$. Generated by the student group Reflux and Chill.

Part 2c: Modification of spectrometer to measure optical properties of perovskite thin films

The Part 2 Discussion Activity has a walkthrough of what to expect from these optical measurements.

- Like discussed in the main text, students should be measuring the %transmittance of their samples (unless the reflectance component can be accounted for). If absorbance is measured instead, students will still observe the line shape that they would expect but should be aware of this source of error.
  - If an instrument setup is available to allow thin film absorbance or diffuse reflectance to be measured, it would be a good addition to this lab to ask students to construct Tauc plots, which plot a transformation of absorbance data to calculate a material's band gap. The transmission and fluorescence measurements allow for an estimation of the optical band gap, but Tauc plots are much more accurate.
- When measuring %transmittance and/or absorbance, sample thickness (and quality) can have an impact on the exact positioning of the observed bandgap. Variations within 10 nm can be expected, especially with higher *n*. Comparisons to research measurements can be found in most of the review articles cited on page S6.
  - If samples are too thick, then it's possible that no light may be transmitted. Overall, we found that this was an uncommon issue. In the beginning, we specifically did not instruct students about the thickness of the films because we wished for them to have the troubleshooting opportunity if the films they deposited initially were too thick.
- For fluorescence measurements, the spectrometer used must have an excitation light source at higher energy than the bandgap. Our spectrometers had a 405 nm LED source as well as a 500 nm LED. While the 500 nm source was also higher energy than the bandgap, it was too close to the expected fluorescence of 525 nm for n = 1 lead iodide perovskites to not drown out the fluorescence signal. Therefore, we encouraged use of the 405 nm source.
  - A longpass filter could also be inserted between the sample and detector to cut out the incident light from the excitation source. Due to the width of the PL spectrum, it is suggested that the cutoff is at least 30 nm lower than the expected peak excitation (*i.e.,* for an n = 1 perovskite with absorbance ~520 nm, the filter cutoff wavelength should not be longer than 490 nm).

- For higher *n* samples, it is possible to make drop casts that contain multiple *n* phases. If a sample is mixed *n* phase, multiple transitions may be observed in the %T spectrum, corresponding to each *n* phase. However, for fluorescence measurements, only one peak might be observed. This is due to charge funneling where charges from the different *n* layers all move the layer with the lowest energy before recombining (*i.e.* the LED principle discussed on page S3)
- There is a slight shift in energy between the transmittance transition and the photoluminescence peak maximum. This is attributed to the loss of some energy of the excited electron through lattice vibrations (phonons) and other mechanisms, leading to a slightly lower energy photon being released than the energy that was absorbed. More can be read in this review by Strauss and Kagan (*J. Phys. Chem. Lett.* **2018**, *9*, 1434-1447)

Additional Instructor Notes:

- In the student groups, it was suggested that members become an "expert" in one of the three parts and focus on collecting that data and reporting it to the group. While this did make lab time efficient, it was noticed that students were weaker in content knowledge in the sections that they did not contribute as heavily to.
    - This was also noticed during the lab worktime, as group members would not take on additional tasks beyond their "role".
- TA/instructor support for students in the lab was very important for student buy-in and attitude. Instructors should be knowledgeable enough to give practical advice for troubleshooting issues in the lab as well as explaining conceptual reasons for outcomes/observations.
- Training students on how to remove and use gloves when working with lead should be performed prior to any of these experiments.

4. Notes on Alternative Experiments

Tuning the crystal structure

There are hundreds of variations on the 2D perovskite crystal structure. There are many options besides the crystals reported here that can be used.

Some considerations for choosing new starting materials:

- While lead is a toxic metal and there are many conditions for its safe handling, the other options for B-site metals, tin and germanium, are very prone to oxidation from their +2 to +4 form. That makes these experiments difficult when the perovskite can degrade within hours. In addition, while the HI-based synthesis allows for some alleviation from oxidation, the DMF based synthesis will be nearly impossible without dried solvents inside a glovebox (the solution will turn brown and oily with oxidation products). One other option is to investigate double perovskites, which use two B-site metals with +1 and +3 charges that alternate positions. The most common double perovskites use Ag^{+} and Bi^{3+}.
- Changing either the B-site or X-site ions, will change the complexation and their absorbance peak position. There are reports on what the absorbance of these complexes are, but they were not extensively tested by us. An example of the absorbance data for the lead bromide complex can be seen in Figure S6, showing that the lead bromide complex can be observed at approximately 301 nm. We should note that substituting the B-site metal substantially shifts the absorbance wavelength of the B-halide complexes. For example, the tin-iodide complex is theoretically calculated in the range of 200-275 nm, which is well below what the typical student spectrometer can measure (*J. Phys. Chem. A* **2023**, *127*, 4463-4472).
- Be aware of additional toxicity concerns of new spacer cations, especially for those in an amine form, instead of an ammonium salt.
- Students could explore alloying perovskite precursors. For example, mixing BA and HA spacer cations in solution can result in $(BA)_x(HA)_{1-x}PbI_4$ crystals. Alloyed perovskites are reported to have both similar properties to their individual constituent phases, and unique properties of an alloyed phase.

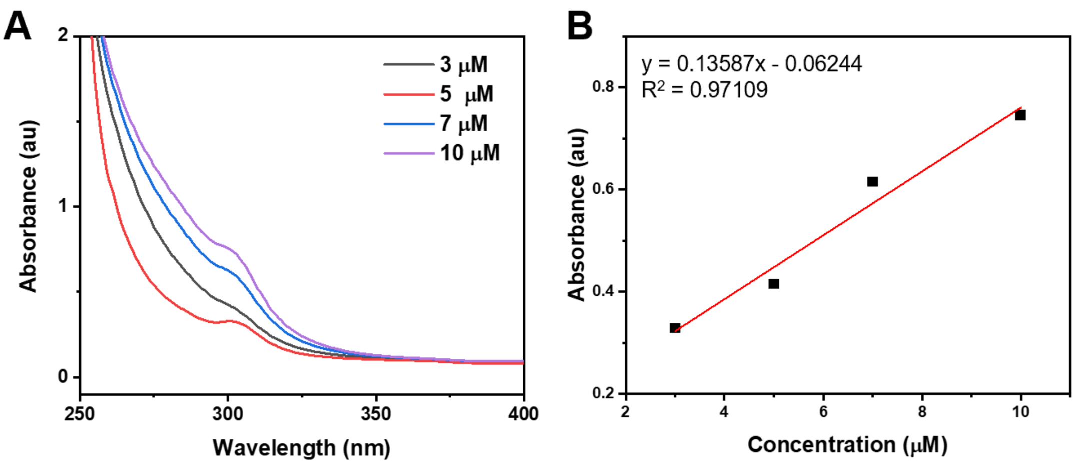

Figure S6. Example of absorbance for lead bromide complex. A) Absorbance spectra for various concentrations of $PbBr_2$ in HBr. B) Calibration curve based off peak absorbance at approximately 301 nm.

Alternative solvents

Most polar organic solvents can be used to grow halide perovskites. Students can explore how concepts like solvent polarity influence perovskite solubility. Most common ones include DMF, dimethyl sulfoxide (DMSO), γ-butyrolactone (GBL), isopropyl alcohol (IPA), and acetonitrile (ACN). Nonpolar solvents, such as toluene, are typically used as antisolvents, or solvents used to force crystals out of solution. As shown in the lab, solvent used can have large effects on solubility and morphology. However, we did not measure the UV-vis absorption for these options so cannot say if that portion of the lab will work exactly as intended.

Powder x-ray diffraction (PXRD)

PXRD is a very common measurement for 2D perovskites as it can be used to determine the interlayer spacing. Patterns of all perovskites used in this experiment can be viewed in Figure S7.

- Typical diffraction peaks for 2D perovskite samples are along the (00*l*) plane, but should be confirmed by examining the out-of-plane direction of the crystal structure.
- Interlayer distance variations are seen between the four n = 1 spacer cations (orange traces), particularly (4AMP)PbI_2 which has the shortest interlayer distance due to being a DJ perovskite.
- The three n = 2 perovskites (red traces) have similar peak spacing since they all used the same spacer cation, HA. The use of different A-cations may have very small effects

on interlayer spacing that can be viewed when zoomed in, but the effect is not as apparent as the spacer cation difference.

- Interlayer spacing variations can be seen between $(HA)_2(MA)_{n-1}Pb_nI_{3n+1}$ (n = 1-3) as the interlayer spacing gets larger
- The black $(HA)_2(MA)_2Pb_3I_{10}$ trace shows some impurities of other n phases with minor anomalous peaks.

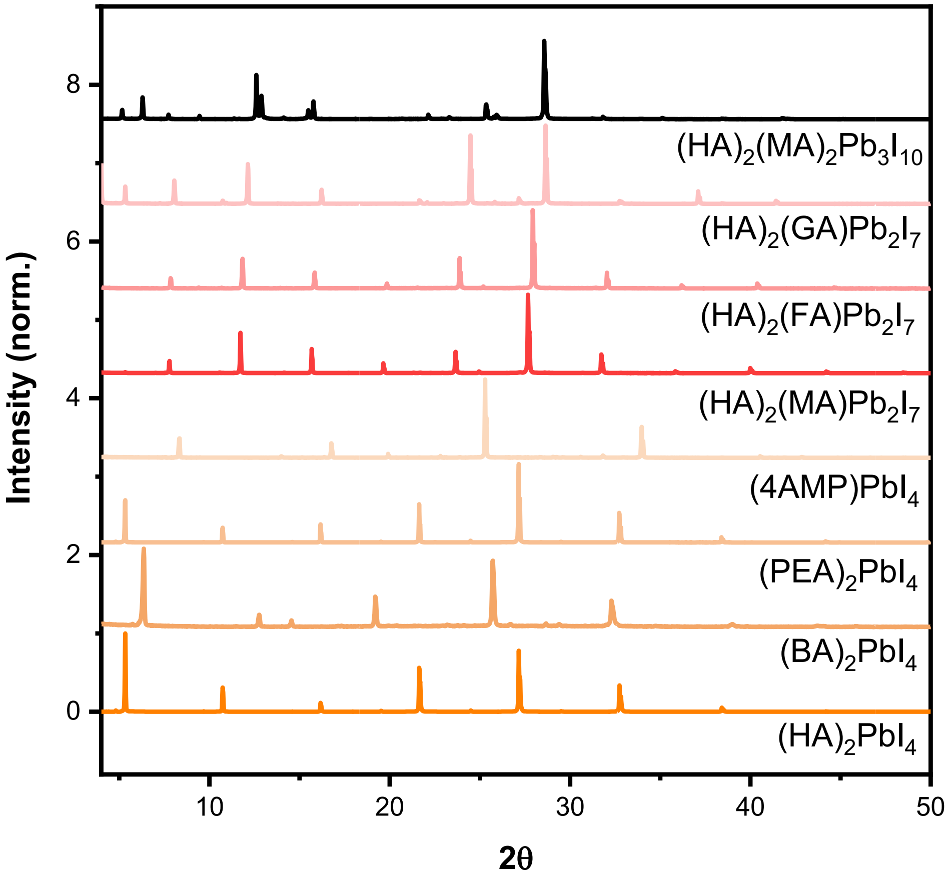

Figure S7. PXRD of various perovskite samples used in this lab.

ICP(-MS or -OES)

Inductively coupled mass spectrometry or optical emission spectroscopy (ICP-MS or -OES) can be used to measure the elemental content of a sample, so can be used for Pb quantification instead of, or in conjunction, with the UV-vis absorption method.

- We confirmed that a calibration curve can be made with PbI_2 standards (Figure S8)
    - Measurement taken on Shimadzu ICPMS-2030

- Samples were primarily diluted with 1% nitric acid. However, hydroiodic acid, even in a small quantity, can be damaging to the instrument.

A

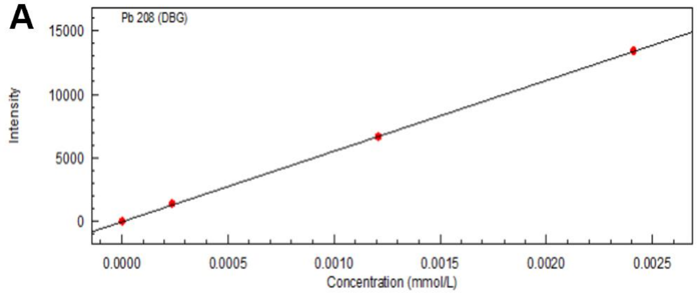

B

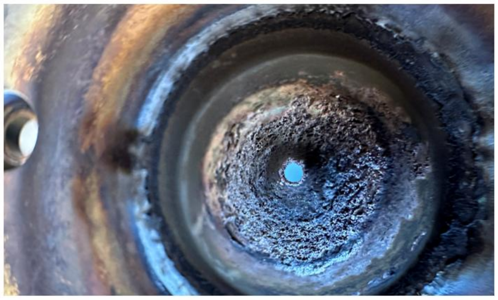

Figure S8. A) Calibration curve of Pb^{2+} concentration collected from standard solutions on ICP-MS. B) Damage to ICP-MS copper cone from HI.

5. Notes on Learning Objectives and Outcomes

Success for the listed learning objectives (LOs) was evaluated through their submission to the Part 1 preparation activity, a midpoint check after Part 1, and final oral presentation that took place at the end of the semester. Students were given a 25 minute time slot consisting of a 15 minute presentation with 10 minutes to answer questions.

Table S2. Breakdown of learning outcomes and tabulation of student success.

LO	Description of and requirement to fulfill LOs	Percentage of student groups
1. Describe how Pb complexation chemistry allows for measuring Pb concentration using a spectrophotometry method, and how solvent choice affects solubility.		
1a.	Describe series of Pb-I complexes that form in solution	100%
1b.	Identify which complexes are visible through UV-vis spectrophotometry	15.3%
1c.	Measure Pb concentration in a 57 wt% HI solution (at normal ratio with H_3PO_2) and a mixed $HI:H_2O$ solution	100%
1d.	Share measured concentrations with the class to show solubility trend with decreasing acidity.	84.6%
2. Optimize the synthesis of a variety of 2D perovskite crystals and characterize through optical microscopy.		
2a.	Successfully synthesized control $(HA)_2PbI_4$ perovskite sample	100%
2b.	Attempt to synthesize additional perovskite crystals based on design variable chosen (If a synthesis attempt was unsuccessful, describe use of Le Chatelier's principle to modify synthesis)	100%*
2c.	Use optical microscopy to observe color and morphology of perovskite crystals	100%
3. Use UV-vis spectrophotometry to measure and quantitatively compare the concentrations of leftover Pb in different perovskite precursor solutions. Propose a relationship between solvent choice, solubility, and perovskite crystal growth.		
3a.	Complete UV-vis measurements of precursor solutions	100%
3b.	Show some comparison between leftover Pb concentrations	100%
3c.	Connect the solubility trends to observed solvent properties or perovskite growth	38.5%

4. Design, modify, and test instrumental and experimental parameters for measuring optical properties of perovskite crystals, such as transmittance and photoluminescence. Connect measured optical properties to the expected perovskite crystal structure.		
4a.	Consider the design principles of spectroscopic equipment to figure out how to measure thin film sample in a cuvette-based set up, for both transmittance and photoluminescence	100%
4b.	Successfully measure transmittance spectra (absorbance also accepted with explanation)	100%
4c.	Attempt to measure fluorescence spectra.	100%
4d.	Connect the band edge energy in transmittance spectra to the expected bandgap from the crystal structure	46.2%
5. "Scientifically communicate via a final presentation the results of their experiments"		
5.	Final presentation grade (Assessed by a panel of 5 reviewers consisting of course staff and teaching assistants)	Average: 82%

*While students all fulfilled the learning objective for synthesizing their perovskite crystals, some crystals are more difficult to synthesize than others. For instructor knowledge, the percentage of student groups that were able to successfully synthesize each crystal is listed in table S3. Note that we count success as some synthesis was successful, whether in single crystal vs film or HI vs DMF.

Table S3. Student success in synthesizing each perovskite crystal

Crystal	Number of groups attempting synthesis	Number of groups successful	Percentage of groups successful
$(HA)_2PbI_4$	13	13	100%
$(BA)_2PbI_4$	5	5	100%
$(PEA)_2PbI_4$	5	5	100%
$(4AMP)PbI_4$	5	5	100%
$(HA)_2(MA)Pb_2I_7$	8	8	100%
$(HA)_2(FA)Pb_2I_7$	3	2	66%
$(HA)_2(GA)Pb_2I_7$	3	2	66%
$(HA)_2(MA)_2Pb_3I_{10}$	5	4	80%

6. Additional Suggested Resources for Students and Instructors

Accessed on November 4th, 2025

Videos explaining semiconductors:

- "What is a semiconductor?" https://www.youtube.com/watch?v=gUmDVe6C-BU
    - Practical demonstration of semiconducting properties
- "Band Gap and Semiconductor Current Carriers" https://www.youtube.com/watch?v=N8MuD_xu6L4
    - How electrons flow in a semiconductor material
- "What is a semiconductor?" https://www.youtube.com/watch?v=YPFk-0CcWgI
    - Chemistry of traditional semiconductors (silicon doping)
- "Semiconductor Manufacturing Process Explained" https://www.youtube.com/watch?v=Bu52CE55BN0&list=PL9jh-CW6ZfSW3Ehx-rTy60sKwZ5_ezsZ7&index=10
    - Industrial manufacturing process of semiconductor chips
- Materialism: Episode 59, Photovoltaic Materials (36:00 for perovskite discussion) (Podcast)

Videos explaining perovskites:

- "Everything you ever wanted to know about perovskite" https://www.youtube.com/watch?v=5oZWLKSDVBk
    - Overview from the natural mineral to near-current research and applications
- "Introduction to Perovskite" https://www.youtube.com/watch?v=0CDQBaQRHFY
    - Why perovskites are of interest to the solar cell community

7. Full Course Schedule

Week	Lab (TR)	Lecture (MW)	Discussion (F)
1	***No Classes (Go through Course Orientation)***	Introduction, Units & Conversions	Class Charter
2	Check In/Good Lab Practices (Day 1)<br>Good Lab Practices (Day 2)	Significant Figures, Error Propagation	HCl & ASA Pre-lab Preparation
3	Preparation of a Standard HCl Solution<br>Determination of %ASA in a Mystery Tablet (Day 1)	Error Propagation, Statistics	Project Lab: Group Charter
4	Determination of %ASA in a Mystery Tablet (Day 2)<br>Spectrophotometric Determination of Iron	Statistics, Spectrophotometry	Project Lab: Discussion Activity (Part 1)
5	Project Lab: Day 1 (Solubility) (Planning at Start)<br>Project Lab: Day 2 (Solubility)	Spectrophotometry	Lecture Support (Emission)
6	Project Lab: Day 3 (Solubility)<br>Project Lab: Day 4 (Solubility)	Equilibria, Acid-Base	Exam 1 Preparation
7	*Make Up Lab Day*<br>***Exam I (1:20-3:20 PM)***	Acid-Base	Fluorescein Pre-lab Preparation
8	Project Lab: Data Work Up (Solubility)<br>Fluorescein (Day 1)	Systematic Treatment, Project Lab Introduction	Project Lab: Discussion Activity (Part 2)
9	Fluorescein (Day 2)<br>Project Lab: Planning Day (Instrument Modification/Band Gap)	Alpha Fractions, Buffers	Project Lab: Time to Finish Planning Assignment
--	***Spring Break***		
10	Project Lab: Day 1 (Instrument Modification/Band Gap)<br>Project Lab: Day 2 (Instrument Modification/Band Gap)	Systematic Treatment, Titration Curves	Lecture Support (Systematic Treatment)
11	Project Lab: Day 3 (Instrument Modification/Band Gap)<br>Project Lab: Day 4 (Instrument Modification/Band Gap)	Activity, EDTA	Exam 2 Preparation
12	*Make Up Lab Day*<br>***Exam II (1:20-3:20 PM)***	Redox	Project Lab: Group Meeting Presentation
13	Hardness of Water<br>Silver Electrode of Equilibria	Electrochemistry	Project Lab: Final Presentation Worktime
14	Introduction to Liquid Chromatography<br>***Project Lab: Final Presentation***	Chromatography	***Final Presentations (Extra Time Slots)***
Finals Week	***Exam 3 (during Final Exam period 5:05-7:05 PM)***		
