# Supplementary material for: Shining Light on Halide Perovskites: Teaching Analytical Chemistry Using Flexible, Inquiry-Based Experiments
Source: J Chem Educ. 2026 Feb 19;103(3):1480–90. doi: 10.1021/acs.jchemed.5c00906 (PMC12980825; doi:10.1021/acs.jchemed.5c00906)
Supplement: Supplementary file 3 [file ed5c00906_si_005.docx]

Shining Light on Halide Perovskites: Teaching Analytical Chemistry Using Flexible, Inquiry-Based Experiments

Kristel M. Forlano, Eliana Bernat, Pamela Doolittle, Dominic Colosi, Song Jin*, Amanda Rae Buchberger*

Department of Chemistry, University of Wisconsin – Madison, Madison, WI, 53706, United States

*Email:

amanda.buchberger@wisc.edu

jin@chem.wisc.edu

**Lab Manual - Part 1**

**Chemistry 329 Laboratory Project –**

**Part 1: PbI_2_ Solubility**

This project was originally developed by Pamela Doolittle, Kristel Forlano, Eliana Bernat, Amanda Buchberger, and Song Jin for Chem 329 Spring 2024. Experiments modified for Chem 329 Spring 2025.

**BACKGROUND**

Materials chemistry and engineering is a bedrock to our world today. One area that is very important in today’s world is electronics and semiconductors. You may have heard of semiconductors in the form of semiconductor chips, which are used in practically every modern technology, from cell phones and computers to household appliances and medical instruments. In addition, semiconductor materials can be found in solar cells and light-emitting devices (LEDs). If you are interested in seeing more about the applications of semiconductors, check out the videos linked on the Canvas project lab page. We will discuss how semiconductors work in Part 2 of this project lab and you will get to measure the properties that make them functional for these applications.

Most semiconductors are inorganic crystal. A crystal is defined through a repeating lattice of bonded atoms. The most common semiconductor crystal in technologies today is silicon. However, there are downsides to silicon, such as the very high processing temperature (> 1000 K) and the need for extremely high purity and defect-free crystals to be functional. As such, alternative types of semiconductor crystals that match the efficiency of silicon, but can provide benefits to some of the downsides, are highly sought after.

Lead-halide perovskites represent a novel category of semiconductor materials due to their affordability, simple fabrication, and tunable properties. Professor Jin’s group at UW-Madison along with other scientists around the globe study the chemistry important to the synthesis of the crystals, with the goal of developing a cheap, robust, and reliable synthesis that will improve efficiencies in a plethora of applications. Perovskite crystals can be synthesized through mixing of precursor salts at a saturated concentration. Heating the mixture and slow cooling will result in the precipitation of the perovskite crystals. You will synthesize perovskite crystals in Part 2 of the project lab.

In Part 1 of the project lab, you will explore the solution chemistry important to the formation of perovskite crystals. Perovskite crystals can be synthesized in a variety of solvents, from aqueous to organic. However, the different solvents will cause the crystals to form in different ways. For example, synthesizing perovskites in acetonitrile will result in powder-like crystals. On the other hand, hydroiodic acid (HI) is one of the typical solvents for growing larger crystals. These different growth results can be useful depending on the final application of the perovskite crystals. As such, understanding the solution chemistry of perovskites is vitally important.

Solvents play a major role in precursor solubility. If one of the precursors used isn’t soluble enough in a given solvent, then enough cannot be dissolved to then let perovskite crystals form. One of the precursor salts regularly used for the formation of perovskite crystals is PbI_2_. If you recall from learning solubility rules, PbI_2_ is generally considered insoluble in water, with a K_sp_ = 7.9x10^-9^. However, PbI_2_ is soluble in aqueous hydroiodic acid. As K_sp_ is generally only calculated for water, we do not quantitively know how much more soluble Pb is in HI than in water. Soluble Pb can be measured through UV-vis spectrophotometry, as Pb coordinates with iodide to form [PbI_3_]ˉ in solution, which absorbs light at approximately 370 nm. In this lab, you will quantitatively determine the solubility of PbI_2_ in HI, and compare it to the solubility of PbI_2_ in H_2_O.

*Notes on HI*

HI is a fairly expensive solvent and can oxidized easily (which forms I_2_). Because of this, we will do the following:

- HI will ALWAYS be mixed with H_3_PO_2_ in a 1:1 ratio. Hypophosphorous acid (H_3_PO_2_) stabilizes HI against oxidation. Throughout this lab, assume that all HI solvent will be mixed.
- Recap your HI vials after withdrawing the solvent you need.
- If your HI solvent (with the included H_3_PO_2_!) is brown, this indicates the presence of I_2_. To reduce the I_2_ back to HI, heat the solvent at approximately 100 °C and swirl it around some. The color should fade to pale yellow/clear. Let your TA know if it doesn’t.
- The scale of your planned experiments should be 1-2 mL. You do not need more than 2 mL of your calibration standards to measure absorbance in a cuvette, and any intermediary solutions you make while diluting the stock PbI_2_ solution to your calibration standards can be on a 1 mL scale.
- You should save your calibration standards rather than remaking them to reduce waste.

**Project Deliverables for Part 1**

1. Create a calibration curve for the quantitative analysis of lead iodide in an HI/H_3_PO_2_ solution mixture
2. Determine the maximum solubility (mg/mL) of PbI_2_ in HI/H_3_PO_2_
3. Determine the maximum solubility (mg/mL) of PbI_2_ in HI/H_3_PO_2_ with either 25%, 50%, and 75% H_2_O.
   1. Your assigned mixture will be shared in lab by your TA.
4. [Share](https://uwprod-my.sharepoint.com/:x:/r/personal/pssemrad_wisc_edu/Documents/03_Analytical%20Project%20Documents/Perovskite%20Project/Spring%202025/SolubilityConstantCollection-PervoskiteProject-Spring2025.xlsx?d=w97df06ba0b61427a97ef2ae74b2dd66c&csf=1&web=1&e=4hhwDo) your determined solubilities with the class, and perform statistical analysis on class wide data to come to an understanding on PbI_2_ solubility trends

*Pro Safety Tips*

- Be very careful handling the reaction matrix mixture. The acids will burn skin and discolor skin and clothing. Lead is toxic. Wear full PPE, including gloves, googles, and your lab coat at ALL times while handling Pb and acid solutions. *Those not following safety procedures will be asked to leave lab for that day.*
- A neutralization solution will be provided for acid spills. Ensure you know where this is. All acid-containing solutions should be neutralized before disposal.
- **All lead containing solutions, reagents, and products must be disposed of in the specially designated waste containers.**

**PRELABORATORY EXERCISES**
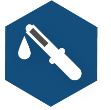


*Below is a list and short description of all pre-lab assessments you will complete.*

**Develop a group charter.** Work with your classmates to develop a document that outlines group expectations. See Canvas for deadlines.

**Complete discussion activity.** Work with your project group to complete a conceptually-focused worksheet. You will need to finish this before moving onto your draft procedure. See Canvas for deadlines.

**Complete a pre-lab quiz.** This is an individual activity to ask for questions you have that we will share with experts later.

**Develop a draft procedure.** The first lab period may be used to plan with your group. You will need to get your TAs approval of your procedure before you enter lab. If prepared, you can enter lab on the planning day. The questions below will help you to develop this procedure.

At minimum, you should have (1) a plan for making a standard calibration curve; and (2) a dilution plan for your saturated PbI_2_ solutions.

Before entering lab, your TA will ask you the following questions:

- What are the concentrations of the standards you plan to make for your calibration curve?
- Approximately, what volume of HI:H_3_PO_2_ mixture do you plan to use?

Ideally, your procedure would be formatted step-by-step. There should be enough detail that someone else could set up your experiment without asking a lot of questions. Answers to the questions below may take additional pages**.**

Remember, though, that your design proposal is the starting point for your experiments. Once your experiments are underway, you will likely need to adjust and refine your procedures. Experiments rarely work the first time exactly as planned! Problems occur and new questions arise. You will modify your experiments to solve the problems and/or answer the questions that arise. You won’t be asked to submit your initial procedure, but, you will need to submit your FINAL version. So, make sure you are keeping track of what changes you make.

**First, let’s consider some practical aspects of the experiment.**

1. Look up the [SDS](https://researchguides.library.wisc.edu/c.php?g=178138&p=1170516) (Safety Data Sheets) for all the chemicals being used in this experiment. Use the link to learn how to search for this information; usually, a good place to start is vendor websites! Because of the danger of working with lead metal powders, you will be given stock solutions of PbI_2_ for Part 1 of the project lab. You should understand the different safety hazards between metal powder and solution-based lead. You CAN handle both safely, with the proper precautions!
2. Come up with an initial plan for preparing (and measuring) a standard calibration curve, which is how you will quantitatively monitor concentrations of the Pb^2+^ in solution. Some things to consider/help you plan:
   1. The [PbI_3_]^-^ complex absorbs strongly (~360 nm), so the range of your calibration curve will need to be very dilute or the detector will not receive any light through the solution in the wanted wavelength range (even if visibly your solution looks transparent). Aim for your MOST concentrated standard to be around 100 μM. If you feel like you need to add points above this concentration to your curve, this can be done during the experiment.
   2. Keep in mind that you will want to dilute all your standards in the 50:50 HI:H_3_PO_2_ mixture. Minimize waste as much as possible (*i.e.* keep volumes in the 1-2 mL range).
   3. In the end, you will want to have at least 4-7 standards (plus a blank) to analyze the soluble lead in solution using PbI_2_ that lie within the optimal range of absorbance for quantitation. Once you figure out a good starting range, you can add more to define the dynamic range better by adding points in-between.
   4. A good strategy for defining the range of a standard curve (starting with 100 μM as the highest standard, remember) is to do step wise dilutions down by a factor of 2, then 5, then 2, etc. This helps you cover multiple factors of 10.
3. All groups will determine the solubility (mg/mL) of PbI_2_ in HI:H_3_PO_2_. Your TA will also assign you to determine the solubility of PbI_2_ in a mixture of HI and water (either 25%, 50%, or 75% H_2_O). This section of the lab will be more difficult to make a concrete plan for because some steps depend on results of previous ones. However, you should come up with a plausible procedure based on the following points:
   1. To determine the solubility of PbI_2_, you need to make a *saturated solution*. What is a saturated solution? How do you make one? Why is it important that the solution is saturated? To minimize working with lead powders in this portion of the project lab, you will be given saturated solutions premade from the stockroom. However, you should understand how they are made.
   2. To determine the concentration of Pb in your saturated solution, you need to measure the absorbance. The saturated solution will likely have a much higher concentration than any stock solutions you made to create your calibration curve. Create a plausible dilution plan assuming ~700 mg of PbI_2_ is needed to create a saturated solution. (Note: This is not an accurate amount, but is intended to give you an idea of the scale of dilutions needed.)
      1. Revisit this planning step after making your saturated solution in lab. What amount of PbI_2_ was required to make a saturated solution? How does this compare to the amount of PbI_2_ used to make your calibration curve standards? Do you need to adjust your dilution plan?
   3. How will you create a saturated solution in your HI:H_3_PO_2_:H_2_O mix? How does the amount of PbI_2_ needed differ from the amount needed for the HI:H_3_PO_2_ mix? What solvents will you use to dilute this saturated solution?
4. After receiving the class data for the solubility of PbI_2_ in the water:HI mixes, you will then need to perform statistics to understand the trends observed. In particular, you likely have learned about tests for 2 populations. Investigate (via Google or other resources) other statistical tests or modifications you will need to make for this study. Specifically, you will have 4 different populations you are comparing (including the mixes and pure HI). You will also want to think about how you will display this in a figure! Viewing how the data will be shared back (see below) may be helpful in your planning.

**EXPERIMENTAL**
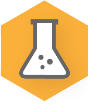


Your group is responsible for time management during the three or four days dedicated to experimentation, although you can find suggested progression of experiments in this text (suggested timeline shared below). In planning your time, work to finish up experimentation by about 5 pm each day, so you’ll have plenty of time for cleanup. **Keep in mind the disposal of all lead containing waste should be placed in the appropriately labelled containers.** Standard PbI_2_ solutions can be saved and reused for subsequent lab periods.

Partial list of what will be provided for you (additional resources provided by request to the stockroom and lab director):

- 0.1 M PbI_2_ stock (*accurate molarity will be shared by stockroom*) dissolved in 50:50 v/v of 50% HI: 50% H_3_PO_2_
  - Can be checked out from the stockroom in 3 mL batches.
- Saturated PbI_2_ solutions in 100%, 75%, 50%, and 25% HI/H_3_PO_2_ (with other percentage of H_2_O)
  - Checked out from the stockroom as 1 mL batch. You will be told how much PbI_2_ was used to create the saturated solution.
- Reaction matrix mixture 50:50 v/v of 50% HI: 50% H_3_PO_2_

Examples of what the stockroom has:

- Volumetric Flasks (10, 25, 50 mL)
- Volumetric Pipets (0.5 mL; 0.1-1.0 mL)
- Wiretrols (5/10, 25, 50/100 uL)
- Graduated Cylinders (10, 50, 100 mL)
- Morter and Pestle
- Spice Grinder
- Vortex Mixer
- Sonic Cleaner
- Centrifuge
- Furnace *(to ash samples)*
- pH Paper & Probes
- Parafilm
- Aluminum Foil
- Plastic Wrap
- Vacuum Filtration Setups (250, 1000 mL)
- Freezer/Fridge
- Bottles (125, 250, 500, 1000 mL)
  - Can be checked out from the stockroom in 20 mL batches
  - HI has a 57 wt% purity prior to dilution.
- 2-dram vials (~7 mL)
- Red Tide UV-Vis spectrometers (measures 200-850 nm) that connect to Vernier Technology (*i.e.,* LabQuests) already in lab
  - We do not have enough for each group to have their own, so make sure you plan accordingly how to make your solutions to minimize your time needed with the spectrophotometers.
- Quartz cuvets

**Overall Project Timeline:**

*Friday, February 14^th^ (Discussion):* You will complete a prelab activity that will introduce you to solubility and complexation topics, especially related to PbI_2_. While solubility has not yet been discussed in lecture, most of the topics should be familiar from your general chemistry course. Work together with your group, classmates, and TA to thoroughly understand this material, as you will see the connection to your lab results. You will need to submit this before starting on your procedure.

*Tuesday February 18^th^ (Lab):* The project introduction day serves as time to meet with your group members to discuss the science and come up with an initial set of methods for measuring and quantifying the soluble lead species. Work with group members to finish the questions related to the “Planning” assignment (*see pre-lab above*), which will help in developing an experimental plan for your project. Consider using collaborative software to have each contribute to the assignment. Be sure to address the various duties and tasks necessary to complete the deliverables and map out a strategy to achieve the results. Try to get everyone in the group to participate in the discussions. It is important to build good team skills, not only for this course but also for your future. Good team skills are one of the most important qualities that employers look for and one of the keys to success. Healthy group discussions should have advocates, skeptics, cheerleaders, strategists, dreamers, *etc.* Sometimes individuals play one of the roles, but the best groups have each person playing all the roles (at different times). Refer to your Group Charter for more information*. If you complete all prelab activities and have a planned procedure, you may begin working in the lab on this day after being checked off with your TA.*

*Thursday, February 20^th^ (Lab):* Finish any procedure planning and check off with your TA to enter lab. Develop and implement a control experiment for measuring lead using wet chemistry methods through the use of spectrophotometry. The TAs will be available to answer questions. Regents and equipment may be checked out from the stockroom.

*Tuesday/Thursday February 25^th^ and 27^th^ (Lab):* Continue working on experiments to quantitatively determine the concentration of Pb in solution. Share your determined solubilities with the class by entering your values into an Excel sheet (link posted on Canvas) for your saturated solutions. Perform statistical analysis on the class wide results to determine a trend of PbI_2_ solubility in the HI/H_2_O solvent system.

**Your group should continue to keep track of your research progress in your laboratory notebooks.**

**RESULTS/CALCULATIONS**
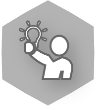


*Below is a list and short description of all post-lab assessments you will complete. More details and expectations are posted on Canvas.*

**Lab notebook pages (*Due March 18^th^ at 1:20 PM, 5 points*).** The notebook pages should be from one group member; that is, don’t hand in duplicate pages with the same information from everyone in the group. We do not expect your notebook pages to be works of art, so no worries if there are mistakes, things crossed out, or incomplete analysis. Notebook pages do have a point value attached to each submission. Pages should be dated and list all group members. Show a progression of work over multiple days. EACH DAY:

1.  State your objective/purpose;

2.  Outline your plan procedure;

3.  Show thinking + doing;

4.  Log results/explicitly calculate things;

5.  Provide a short reflective summary and the next steps for each entry.

Even if you're simply meeting with group members, capture that meeting in your notebook.  Think of this as a way to take minutes of your meeting, and also a means to capture the contributions and ideas of all group members.

**Peer Feedback (*Due March 18^th^ at 1:20 PM, 15 points*).** Provide mid-project peer feedback for all your group members (including yourself). See Canvas for link to Google Form.

**Lab Report (*Due March 18^th^ at 1:20 PM, 20 points*).** Each group should submit the following documentation in a single PDF:

- Your finalized procedure for this experiment. This can be in bullet point form with any useful tables or calculations. Make sure it is specific enough that someone else could read the procedure and reproduce it.
- At least one example (*e.g.,* most concentrated standard) full wavelength spectra of the lead iodide solution.
- Example calibration curve for determining the concentration of Pb species in solution. Make sure your axes are well labeled. Include your linear regression equation (y=mx+b) and the R^2^ value.
- A graphic that depicts the results of the solubility comparison of PbI_2_.Think of this as a “capstone” figure that captures the most important part of this experiment. Include the solubility of PbI_2_ in water in some way on the graph along with the 4 other solubilities tested in this project lab.
