# Supplementary material for: Shining Light on Halide Perovskites: Teaching Analytical Chemistry Using Flexible, Inquiry-Based Experiments
Source: J Chem Educ. 2026 Feb 19;103(3):1480–90. doi: 10.1021/acs.jchemed.5c00906 (PMC12980825; doi:10.1021/acs.jchemed.5c00906)
Supplement: Supplementary file 6 [file ed5c00906_si_008.docx]

Shining Light on Halide Perovskites: Teaching Analytical Chemistry Using Flexible, Inquiry-Based Experiments

Kristel M. Forlano, Eliana Bernat, Pamela Doolittle, Dominic Colosi, Song Jin*, Amanda Rae Buchberger*

Department of Chemistry, University of Wisconsin – Madison, Madison, WI, 53706, United States

*Email:

amanda.buchberger@wisc.edu

jin@chem.wisc.edu

**Lab Manual - Part 2**

**Chemistry 329 Laboratory Project –**

**Part 2: Perovskite Synthesis**

This project was originally developed by Pamela Doolittle, Kristel Forlano, Eliana Bernat, Amanda Buchberger, and Song Jin for Chem 329 Spring 2024. Experiments modified for Chem 329 Spring 2025.

**BACKGROUND**

Two-dimensional lead halide perovskites represent a novel category of semiconductor materials with promising applications in optoelectronic device construction, such as solar cells and LEDs, due to their affordability, simple fabrication, and tunable properties. [Professor Jin’s group at UW-Madison](https://sjin.chem.wisc.edu/) along with other scientists around the globe study the chemistry important to the synthesis of the 2D structures, with the goal of developing a cheap, robust, and reliable synthesis that will improve efficiencies in a plethora of applications.

These perovskite structures form using a general formula of (LA)_2_(A)*_n_*_-1_Pb*_n_*X_3_*_n_*_+1._ *(Review the Part 2 discussion activity to remind yourself about what each of these symbols mean, if needed).* Principles of their formation lie in solubility equilibrium. Precursors are mixed in a solvent, with concentrations chosen such that upon heating the mixture, the result is a super saturated solution. Slow cooling of the solution results in a homogeneous precipitation of the products and a formation of the 2D crystals. In your discussion activity, you learned about the crystal structure of perovskites and how their composition relates to their bandgap energy, as shown in **Figure 1**.


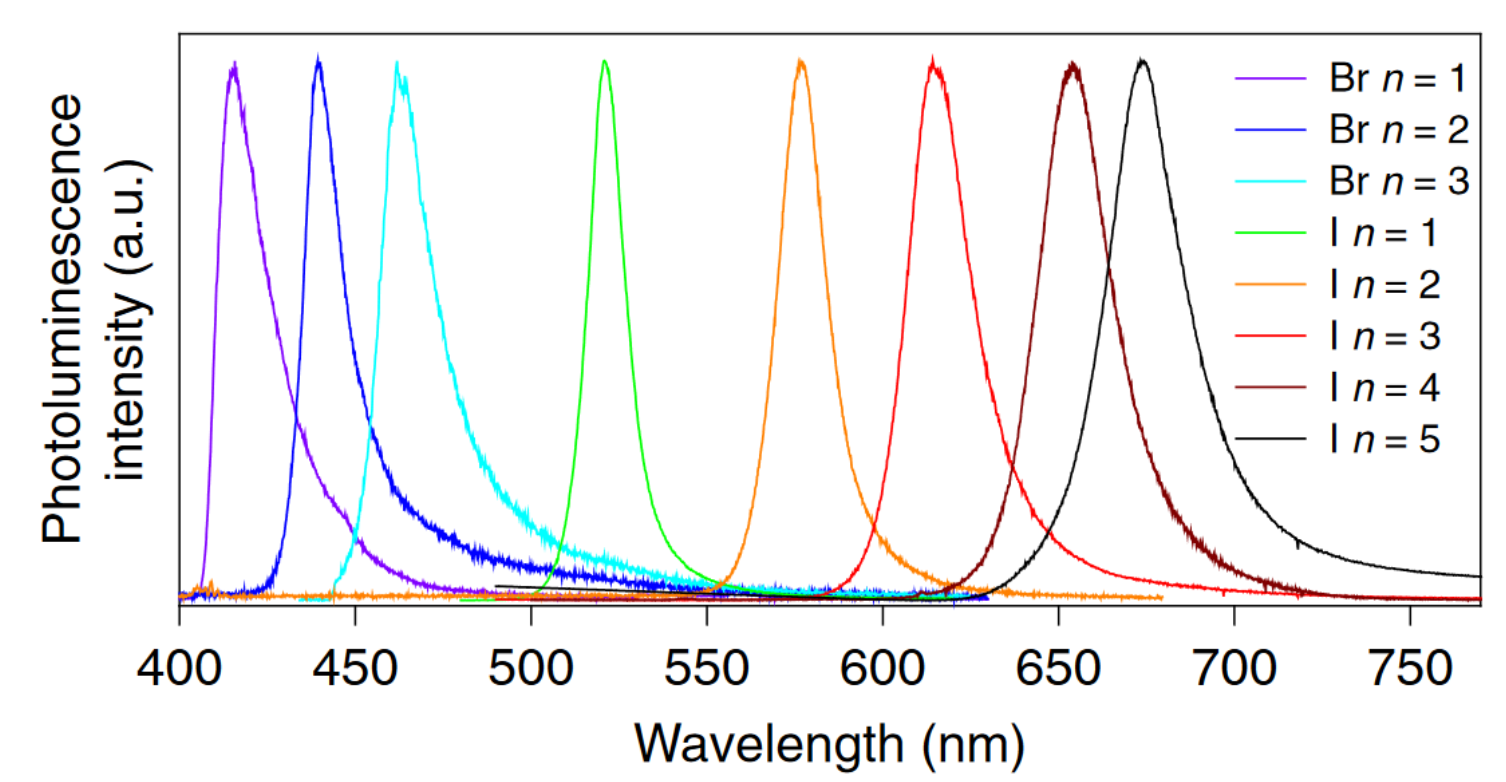


***Figure 1****: Photoluminescence for 2D RP perovskites with various X-site and n number. compositions (Adapted with permission from Pan et. al.^1^ Copyright 2021 Nature.)*

In this second leg of the project lab, you will get to synthesize a variety of perovskites, connect observations on their growth to solubility, and measure the properties that make them semiconductors. Unlike other labs you may have participated in before, there is no one answer or goal that you’re trying to find. Instead, the goal is to develop a holistic understanding of the perovskites, from solubility to growth and properties to applications.

**Project Deliverables for Part 2**

1. Synthesize (*n*-hexylammonium)_2_PbI_4_ perovskite crystals in HI and DMF (dimethylformamide) solutions.
2. Measure concentration of Pb in HI and DMF solutions and compare results to crystal growth outcomes.
3. Observe crystal growth through taking pictures of crystals in the vials and pictures under a microscope.
4. Design, modify, and test instrumental and experimental parameters for measuring optical properties of (HA)_2_PbI_4_ crystals in spectrophotometers, such as transmittance and photoluminescence.
5. Synthesize variety of perovskite crystals following a specific design trend (explained below). Repeat deliverables 2 through 4 for all synthesized perovskite crystals.
6. Present holistic overview of perovskite crystals – synthesis/solubility and properties.

*As a reminder, we are performing real research. Some of these deliverables may have never been performed before at all or at the depth you are going to develop them for our system or application. Be curious, honest, and open to trying new things! If something doesn’t work, that is OK! The important thing is that you TRIED to complete the goals of the lab.*

**General Method for Perovskite Synthesis**

The crystals are grown in 2 dram (~7 mL) vials. The matrix for the solution growth is 1 mL of a 50:50 (v/v) mixture of about 50% by weight HI and 50% by weight hypo-phosphorous acid (H_3_PO_2_) or DMF. Determine the mass for each precursor necessary to create stoichiometric ratios (see prelaboratory exercises for details) of the precursors in the 1 mL of reaction mixture.

Weigh the calculated amounts into a weight boat using the analytical balance. You should be accurate to the second decimal place but it’s okay if the reading from the balance differs from the calculated amount past that. Transfer the reagent from the weigh boat to the reaction vial scraping using a spatula. If you have liquid forms of the compound, transfer an amount of solution that is appropriate to the grams desired. Once all precursors are added to the vial, close and mix, being careful to ensure the cap is fully closed on the vial. Note the salts may not fully dissolve and you’ll observe solid in the final mixture at room temperature.

To dissolve the salt precursors, the vial must be heated. There are several options to choose from to include using the ovens in lab, directly placing the vial on a hot plate set to 100 degrees °C, or by using a hot bath and clamp apparatus (See **Figure 2**). Once the solutions are heated, the salts should nearly or completely dissolve. Look carefully for any small particulate at the bottom of the vial, as the presence of the solid is an indication your reaction mixture may need to be heated a bit longer.

***
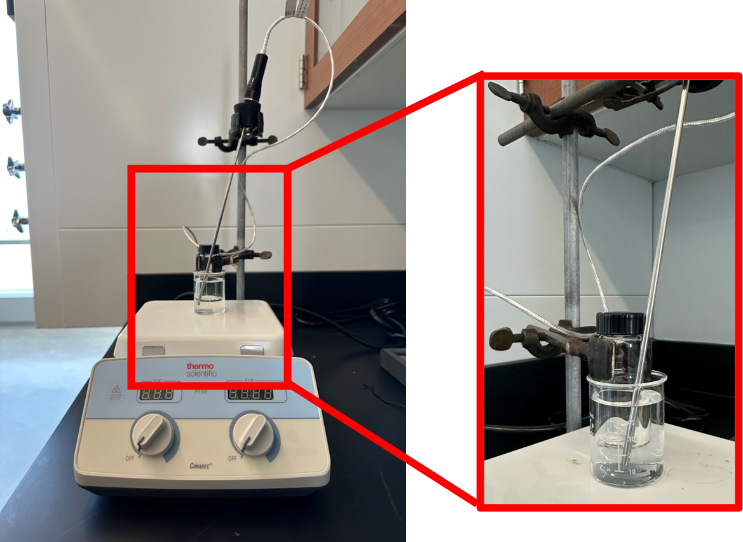
***

***Figure 2****: Example set up for heating mixture.*

*Pro Tips*

- Don’t overheat the reaction mixture as it could compromise the vial!
- Use a towel, thermal glove or cheese cloth to handle hot vials to protect your skin from hot glass.

Once the salts are dissolved, decrease the temperature on the hot plate about 10 °C every 10 minutes. At about 70 °C, you should notice the formation of small crystals around the top and sides of the glass. Continue to decrease the temperature until you reach 50 °C, after which point you can remove the vials from the heat source and let the solutions cool naturally to room temperature. Examples of possible colors for products are illustrated in **Figure 3**. These are the typical colors for lead iodide perovskite crystals and therefore can be used as a visual sign of synthesizing crystals with a targeted *n* number. By shining a black light onto the products, you should observe luminescence per the information provided in **Figure 1.**


***Figure 3****: Example perovskite products from left to right for n=1 (orange), n=2 (red) and n=3 (black). All of these products fluoresce, and the predicted emission wavelengths are provided in Figure 2d.*

**PRELABORATORY EXERCISES**
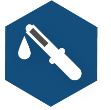


*Below is a list and short description of all pre-lab assessments you will complete.*

**Complete discussion activity.** Work with your project group to complete a conceptually-focused worksheet. You will need to finish this before moving onto your draft procedure. See Canvas for deadlines.

**Complete a pre-lab quiz.** This is an individual activity to ask for questions you have that we will share with experts later.

**Develop a draft procedure** Using the background and the following questions, submit a plan on how to approach Part 2’s deliverables. This procedure will be submitted and feedback given before the first laboratory period.

You should plan to answer/include the below questions in your planning submission. At minimum, you should have (1) a table that includes the chemicals being weighed out, the amount to weigh out, and any notes about their chemical properties/safety considerations; (2) a drawn diagram of how you plan to measure the photophysical properties of the perovskite crystals through modifying the given spectrometers; and (3) a list of all measurements planned to be taken with brief procedures for each.

Ideally, your submission would be formatted in a way that is a step-by-step procedure. The procedure part of your submission should be terse – no more than 2 pages of text outlining the proposed procedures – yet with enough detail that someone else could set up your experiment without asking a lot of questions. Answers to the questions will take additional pages**.** As there are many different experiments, it could also be useful to assign group members to certain parts of the procedure (*i.e.,* while one group member is examining the perovskite crystals under a microscope, another is measuring the transmittance).

Remember, though, that your design proposal is the starting point for your experiments. Once your experiments are underway, you will likely need to adjust and refine your procedures. Experiments rarely work the first time exactly as planned! Problems occur and new questions arise. You will modify your experiments to solve the problems and/or answer the questions that arise. This process will be repeated multiple times over the course of the project.

**First, let’s consider some practical aspects of the synthesis portion of the experiment. (Learning Objectives 1, 2, and 3)**

1. Look up the [SDS](https://researchguides.library.wisc.edu/c.php?g=178138&p=1170516) (Safety Data Sheets) for all the chemicals being used in this experiment. Use the link to learn how to search for this information; usually, a good place to start is vendor websites!
   1. Many of the spacer cations and A-site cations are *hydroscopic*, meaning they react with water. When writing your procedure, make sure to include capping all vials immediately in your procedure, and as an extra precaution, store in a desiccator.
2. ALL GROUPS will begin with synthesizing (HA)_2_PbI_4_ as a model perovskite system (HA = *n*-hexylammonium).
   1. Calculate how much of the precursor salts will be needed to make a 0.1 M perovskite solution. **Table 1** shows an example of how to organize your calculations. You will be making perovskite solutions in both HI/H_3_PO_2_ and DMF.

***Table 1****: Suggested table for organizing information in your lab notebooks (example for n=1).*

| Precursor | Molecular Weight | Stoichiometric ratio for n = 1 | Goal Molarity (M) | Mass (mg) |
| --- | --- | --- | --- | --- |
| n-hexylammonium iodide (HAI) | 229.1 |  |  |  |
| PbI_2_ | 461.0 |  | Stock solution concentration | X |

- 1. You will be given vials with aliquots of 1 mL 0.1 M Pb^2+^ in HI/H_3_PO_2_ to make your perovskite crystals in.
  2. You will be measuring the Pb^2+^ concentration in all perovskite solutions, both acidic and DMF. Using your knowledge from Part 1, plan an approximate dilution scheme to get the concentration of Pb^2+^ in a measurable range.
     1. You will be using a [research-grade UV-vis spectrometer](chrome-extension://efaidnbmnnnibpcajpcglclefindmkaj/https:/jascoinc.com/docs/legacy-product-pdf/v550-60-70spec-sheet.pdf) located on the 7^th^ floor of the North Tower. Dr. B. will accompany group members to demonstrate how the instrument works. We will provide a calibration curve that you will then analyze when you analyze your sample. Talk to Dr. B about signing up, when ready.
     2. Collect data in full spectrum mode. You should then export the data in tab-delimited form for graphing figures later.

**Now, let’s consider some practical aspects of the instrument-modification portion of the experiment. (Learning Objectives 3 & 4)**

1. In order to measure the properties of the perovskite crystals, you will need to deposit them on a substrate (**Figure 4**).
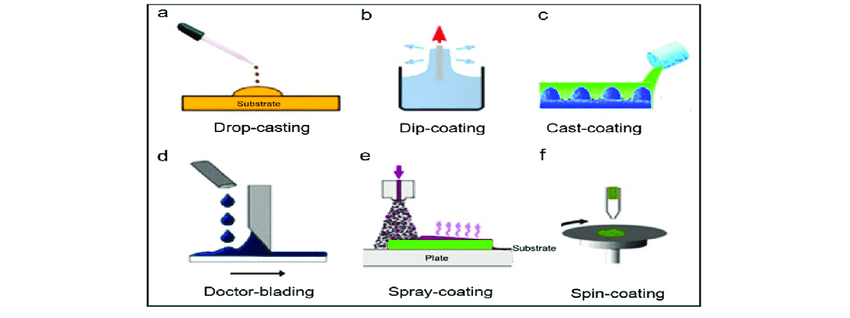


***Figure 4****: Graphic explaining different ways perovskite cystals can be deposited on a substrate. (Reprinted with permission from Ramaiah et. al.^2^ Copyright 2021 Elsevier.)*

Many of these techniques require more advanced techniques or equipment, so it’s suggested that you use the simple dropcasting technique. This technique begins by heating your precursor solution. Heating the solution causes some of the precipitated crystals to redissolve in solution and the solution becomes *supersaturated (meaning more is dissolved than the expected solubility limit)*. A droplet of the supersaturated solution is deposited on a piece of heated glass, and the solution is allowed to evaporate. This leaves behind deposited crystals. If you are interested in exploring other techniques over the course of the lab, you are welcome to try and/or ask about equipment availability!

- 1. When you are ready to try out depositing your crystals, the TAs will demonstrate the dropcasting technique.
  2. You are evaporating off solvents that are NOT GOOD TO BREATH. Therefore, all depositions should be done on hot plates in the fume hoods.
  3. Around 60-80 °C is a good starting point for the temperature that you should have your solution and deposit at. Try out different temperatures and see what you observe change in the crystal growth!
  4. You will be depositing the perovskite crystals on glass slides. However, glass slides are relatively large for our purposes and you’ll likely be wanting them with certain dimensions for placing them in the spectrometer (*Hint! See below!*). You will be shown how substrates, including glass, are cut in lab through scoring with a diamond-tip pen.
  5. You will be able to observe the perovskite crystals through many formats, including under the microscope. You should be making observations on how the crystals form between the acid-based and DMF solutions. Take pictures from the microscope and vials! There will also be ultraviolet flashlights to shine on the crystals to observe the photoluminescence.
     1. One method to observe crystal growth under a microscope is to drop ~5 uL of the heated precursor solution onto a slide placed under a microscope. Because the solution is cooling, this is fine to do outside of a fume hood.

1. To observe the photophysical properties that make perovskite semiconductors, transmittance and photoluminescence measurements should be taken. However, these crystals are not in a liquid form that you can put into a cuvette. You will need to think outside the box on how to use the spectrophotometers that are available to take these measurements.
   1. Draw a diagram of the spectrometer ([Venier SpectroVisPlus](https://www.vernier.com/product/go-direct-spectrovis-plus-spectrophotometer/?srsltid=AfmBOooGL7XdL_GBpU3cFWk5nrkJV2dUKZgQsi0YOUyqzByoy407984U) we have in lab). Your sample is on a piece of flat, 2D glass. For transmittance measurements, how should the sample be placed?
   2. Photoluminescence (PL) measurements are trickier. Draw a diagram of how the spectrometer functions in PL mode. How would the sample need to be placed?
   3. You do NOT need to disassemble the instrument to take these measurements. Everything can be done through modifying the sample placement.
   4. REMEMBER: Collect data in full spectrum mode. You should then export the data in tab-delimited form for graphing figures later.

**Finally, let’s consider some practical aspects of the extra “design” part of this experiment. (Learning Objectives 5)**

1. After gathering a full data set of growing crystals in acids and DMF, including the Pb^2+^ concentration, microscope images, and the photophysical properties of (HA)_2_PbI_4_ via instrument modifications, you will get to explore a novel question across perovskite crystals. Several kits have been made up to explore how changing one precursor component across perovskite crystals changes the growth and properties. You will get to choose with your group what topic you’d like to explore. Read through the following options. Before starting the planning process, sign up your group through the [Excel sheet](https://uwprod-my.sharepoint.com/:x:/g/personal/pssemrad_wisc_edu/EXdtZZyNOUVOrrIL3mOjGNUBrn9ZXfLtMYxxE-5CzqhVPw?e=4jHe6V) to claim the experiment (meaning on the planning day). We have enough supplies to allow for a max of 5 groups per option (except for Option d).
   1. **Impact of spacer cation on solution growth:** For *n* = 1, change the spacer cation to butylammonium iodide (BAI), phenethylammonium iodide (PEAI), and 4-(aminomethyl) piperidine (4AMP).
      1. You will receive 1 g of each spacer cation salt (BAI, PEAI) and 0.5 mL of 4AMP.
      2. Look up and draw in your notebook what each spacer cation looks like. What impacts do you think these different spacer cations might have on the crystal growth?
      3. 4AMP is not an iodide salt and is also a liquid. Think carefully about how you will manage the mass and quantitative transfer.
         1. 4AMP is also a diamine salt, meaning it has an NH_3_^+^ group on both ends of the molecule. Instead of the bilayer of spacer cations that you’ve seen in the RP crystal structure, there is instead only a single layer of spacer cations. This means that the formula for these types of perovskites, also known as Dion-Jacobson (DJ) perovskites, is (LA)_1_A*_n_*_-1_B*_n_*X_3_*_n_*_+1_.
   2. **Impact of *n* number on solution growth:** With hexylamine (HAI) as the spacer, use methylammonium iodide (MAI) as the A-site cation and explore *n* = 2 and *n* = 3.
      1. You will receive an additional 2 g of HAI and 2 g of MAI.
      2. Write out the formulas you expect for each crystal phase.
      3. Once you add in the cage cation, it can be hard to get a pure *n*-phase. It’s quite possible to get mixtures of *n* = 2 and *n* = 3, maybe even higher *n*. Pictures will be essential for here because you can use the color differences (orange for *n* = 1, red for *n* = 2, and black for *n* = 3) to help identify the phases. You may need to try changing your recipe several times to get the crystals you want! Keep in mind, the stockroom will have different concentrations of PbI_2_ stock solution for you to use.
      4. Have a plan on how to change your synthesis recipes in case you don’t get the product you expect. You will have access to 0.1 M, 0.3 M, and 0.5 M Pb^2+^ solutions as the basis for your crystal recipes.
   3. **Impact of A-site cation on solution growth:** Using hexylamine (HAI) as the spacer, use methylammonium iodide (MAI), formamidinium iodide (FAI), and guanidinium iodide (GAI) as the A-site cation and explore *n* = 2 crystals.
      1. You will receive an additional 3 g of HAI, and 1.5 g of all A-site cations.
      2. Look up and draw in your notebook what each A-site cation looks like. Write out the formula you expect for the *n =*1 and *n* = 2 crystals.
      3. Once you add in the cage cation, it can be hard to get a pure *n*-phase. It’s quite possible to get mixtures of *n* = 2 and *n* = 3, maybe even higher *n*. Pictures will be essential for here because you can use the color differences (orange for *n* = 1, red for *n* = 2, and black for *n* = 3) to help identify the phases. You may need to try changing your recipe several times to get the crystals you want! Keep in mind, the stockroom will have different concentrations of PbI_2_ stock solution for you to use.
      4. Have a plan on how to change your synthesis recipes in case you don’t get the product you expect. You will have access to 0.1 M, 0.3 M, and 0.5 M Pb^2+^ solutions as the basis for your crystal recipes.
   4. **Choose your own adventure:** There are many other factors influencing the growth of the perovskite crystals that you can explore, including method development for the best way to synthesize/deposit/*etc.* the perovskites. Many have been mentioned throughout these planning questions. If you have a question you’d like to explore, you can propose this to your TA and the course staff. Here are a few guiding questions if you decide to go this route:
      1. The question you choose should have a systematic trend that you can explore (*i.e.,* changing just the A-site cation or changing just the spacer cation.). If you choose to change too many things at once, then you don’t know what is affecting your system.
      2. The materials list given below incorporates the materials for the suggested experiments above. However, if you have a proposed experiment that would require different materials, you are welcome to ask if they are available. Other perovskite precursors, solvents, etc. may be available to borrow from Prof. Jin’s lab.
      3. If you have a basic idea of a proposed experiment, but don’t know what may be possible or available, ask! If you have a question that you want to explore, the course staff will try their best to provide the materials, equipment, and expertise to let your curiosity run wild.

**EXPERIMENTAL**
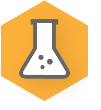


Your group is responsible for time management, although you can find suggested progression of experiments in this text (suggested timeline shared below). In planning your time, work to finish up experimentation by about 5 pm each day, so you’ll have plenty of time for cleanup. **Keep in mind the disposal of all lead containing waste should be placed in the appropriately labelled containers.** Standard PbI_2_ solutions can be saved and reused for subsequent lab periods. Store excess solid material in a properly prepared desiccator.

*Pro Safety Tips*

- Be very careful handling the reaction matrix mixture. The acids will burn skin and discolor skin and clothing. Lead is toxic. Wear full PPE, including gloves, googles, and your lab coat at ALL times while handling Pb and acid solutions. *Those not following safety procedures will be asked to leave lab for that day.*
- A neutralization solution will be provided for acid spills. Ensure you know where this is. All acid-containing solutions should be neutralized before disposal.
- NEW: DMF can penetrate gloves and your skin with short exposure times. It can also carry anything that is dissolved in it, including the Pb^2+^ you will be working with. If you get DMF on your gloves, especially if the solution contains lead, change gloves IMMEDIATELY.
- **All lead containing solutions, reagents, and products must be disposed of in the specially designated waste containers.**

Partial list of what will be provided for you (additional resources provided by request to the stockroom and lab director):

- 0.1 M, 0.3 M, and 0.5 M PbI_2_ stock (*accurate molarity will be shared by stockroom*) dissolved in 50:50 v/v of 50% HI: 50% H_3_PO_2_
  - Can be checked out from the stockroom in 1 mL batches.
- 0.1 M, 0.3 M, and 0.5 M PbI_2_ stock (*accurate molarity will be shared by stockroom*) dissolved in DMF
  - Can be checked out from the stockroom in 1 mL batches.
- Spacer cations:
  - *n*-hexylammonium iodide (HAI)
  - *n*-butylammonium iodide (BAI)
  - phenethylammonium iodide (PEAI)
  - 4-(aminomethyl) piperidine (4AMP)
- A-site cations:
  - Methylammonium iodide (MAI)
  - Formamidinium iodide (FAI)
  - Guanidinium iodide (GAI)
- Reaction matrix mixture 50:50 v/v of 50% HI: 50% H_3_PO_2_

Examples of what the stockroom has:

- Volumetric Flasks (10, 25, 50 mL)
- Volumetric Pipets (0.5 mL; 0.1-1.0 mL)
- Wiretrols (5/10, 25, 50/100 uL)
- Graduated Cylinders (10, 50, 100 mL)
- Morter and Pestle
- Spice Grinder
- Vortex Mixer
- Sonic Cleaner
- Centrifuge
- Furnace *(to ash samples)*
- pH Paper & Probes
- Parafilm
- Aluminum Foil
- Plastic Wrap
- Vacuum Filtration Setups (250, 1000 mL)
- Freezer/Fridge
- Bottles (125, 250, 500, 1000 mL)
  - Can be checked out from the stockroom in 20 mL batches.
  - Plan to only need 1 batch.
  - HI has a 57 wt% purity prior to dilution.
- Dimethylformamide (DMF)
  - Can be checked out from the stockroom in 20 mL batches
- 2-dram vials (~7 mL)
- Access to an appropriate spectrophotometer that can measure in the UV range. *(See part 2c of the prelaboratory planning activity for details)*
- Quartz cuvets
- Glass slides (you’ll need to score and cut to size using diamond scribes)
- Stereomicroscope for observing crystal growth in real time.

**Overall Project Timeline:**

*Friday, March 14^th^ (Discussion):* You will complete a prelab activity that will introduce you to perovskite crystals and semiconductor properties. Work together with your group, classmates, and TA to thoroughly understand this material, as you will need much of this information to begin planning your procedure. You will need to submit this before starting your procedure. See Canvas for deadlines.

*Thursday March 20^th^ (Lab):* This procedure planning day allows you to meet with your group and develop a procedure that will take you through this second part of the project lab. Work with group members to finish the questions related to the “Planning” assignment (*see pre-lab above*), which will help in developing an experimental plan for your project. Consider using collaborative software to have each contribute to the assignment. Be sure to address the various duties and tasks necessary to complete the deliverables and map out a strategy to achieve the results. In particular, this part of the project lab has many components that can be completed simultaneously with group members working on different parts. Assigning group members to different tasks is a great way to be efficient with your time. You will submit your procedure for feedback.

*Friday, March 21^st^ (Discussion)*: Finish working on your procedure. Submit your procedure by the end of this discussion period (12:00 PM) for feedback. *See Canvas for details.*

*Tuesday April 1^st^ (Lab):* Discuss any feedback received and make adjustments to your procedure (should aim for this to take up minimal lab time). Begin synthesis of (HA)_2_PbI_4_. Attempt to deposit the crystals. Make observation of the crystals and take notes on any changes or differences observed. Explore using the microscope to view the crystals. Begin attempting to use the spectrometer to measure optical properties of the perovskite crystals.

*Thursday April 3^rd^ (Lab):* Continue collecting data on (HA)_2_PbI_4_ crystals. After working with the sample and modifying procedures, you may want to write out a “final” procedure that you believe can be carried out across the next perovskite samples you will make. If time allows, begin synthesizing your next sets of perovskite crystals.

*Tuesday April 8^th^ (Lab):* Synthesize your other perovskite crystals. Depending on what samples you are attempting, you may need to fine-tune recipes. Begin taking measurements on your other perovskite samples.

*Thursday April 10^th^ (Lab):* Last project day; today your experiments are with the purpose of completing a compelling story to tell during the final presentation. Finish collecting any data that you think you might need. If you are finished before the end of the lab period, take some time to sit with your lab group and go over all of the data you’ve collected. It’s likely each group member will have different data they’ve collected, so make sure nothing was overlooked!

*Friday, April 18^th^ (Discussion;* ***5 points – see Canvas for more details****):* Prepare a short (5 slides maximum) PowerPoint presentation outlining your story or a portion of your story (as described on Canvas). The exact focus will depend upon the progress in the lab, so stay tuned!

*Friday, April 25^th^ (Discussion)*: This will be an unstructured work time on your slides; please come to discussion and put the final touches on your slides! If you wish to get feedback on your final slides, please send an email to Dr. B by the end of the weekend; she will then give you feedback on Monday, 4/28/2025.

*Thursday, May 1^st^ (Lab) and Friday, May 2^nd^ (Discussion+) (****50 points – see Canvas for more details****):* You should capture all the project deliverables and key considerations in this final presentation. Capture your major data/methods in figures or tables, such as calibration curves, comparison graphs, or methodology workflow. All graphs should include a title, (axes) labels, and units/numbers as appropriate. Try not to overuse tables!

**Your group should continue to keep track of your research progress in your laboratory notebooks.**

**RESULTS/CALCULATIONS**
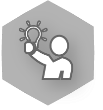


*Below is a list and short description of all post-lab assessments you will complete. More details and expectations are posted on Canvas.*

***NOTE: There are 2 other assessments articulated in the project timeline above.***

**Lab notebook pages (*Due May 2^nd^ at 11:59 PM, 5 points*).** The notebook pages should be from one group member; that is, don’t hand in duplicate pages with the same information from everyone in the group. We do not expect your notebook pages to be works of art, so no worries if there are mistakes, things crossed out, or incomplete analysis. Notebook pages do have a point value attached to each submission. Pages should be dated and list all group members. Show a progression of work over multiple days. EACH DAY:

1.  State your objective/purpose.

2.  Outline your plan procedure.

3.  Show thinking + doing.

4.  Log results/explicitly calculate things.

5.  Provide a short reflective summary and the next steps for each entry.

Even if you're simply meeting with group members, capture that meeting in your notebook.  Think of this as a way to take minutes of your meeting, and a means to capture the contributions and ideas of all group members.

**Peer Feedback (*Due May 2^nd^ at 11:59 PM, 25 points*).** Provide end-of-project peer feedback for all your group members (including yourself). See Canvas for link to Google Form. Please only use your experience in Part 2 to guide your reviews.

**Data Submission (*Due May 2^nd^ at 11:59 PM, 5 points*).** Please submit an Excel sheet that contains the RAW spectrophotometry data (well labeled) using the template provided on Canvas.

**REFERENCES**

(1) Pan, D.; Fu, Y.; Spitha, N.; Zhao, Y.; Roy, C. R.; Morrow, D. J.; Kohler, D. D.; Wright, J. C.; Jin, S. Deterministic Fabrication of Arbitrary Vertical Heterostructures of Two-Dimensional Ruddlesden–Popper Halide Perovskites. *Nat. Nanotechnol.* **2021**, *16* (2), 159–165. https://doi.org/10.1038/s41565-020-00802-2.

(2) Ramaiah, G. B.; Tegegne, A.; Melese, B. Developments in Nano-Materials and Analysing Its Role in Fighting COVID-19. *Materials Today: Proceedings* **2021**, *47*, 4357–4363. https://doi.org/10.1016/j.matpr.2021.05.020.
