# Supplementary material for: Shining Light on Halide Perovskites: Teaching Analytical Chemistry Using Flexible, Inquiry-Based Experiments
Source: J Chem Educ. 2026 Feb 19;103(3):1480–90. doi: 10.1021/acs.jchemed.5c00906 (PMC12980825; doi:10.1021/acs.jchemed.5c00906)
Supplement: Supplementary file 7 [file ed5c00906_si_010.pdf]

Shining Light on Halide Perovskites: Teaching Analytical Chemistry Using Flexible, Inquiry-Based Experiments

Kristel M. Forlano, Eliana Bernat, Pamela Doolittle, Dominic Colosi, Song Jin*, Amanda Rae Buchberger*

Department of Chemistry, University of Wisconsin – Madison, Madison, WI, 53706, United States

*Email:
amanda.buchberger@wisc.edu
jin@chem.wisc.edu

Preparation for Perovskite Project Lab Part I

Purpose: The following activity is meant to help you think about solubility and complexation concepts as applied to Part 1 of the Perovskite Project Lab. This discussion activity must be completed and submitted to Canvas before you enter lab on the project lab days. If you finish this activity early during discussion, it's suggested that you begin drafting a procedure for the project lab beginning on February 18th.

Learning Objectives:

- *Review concepts of solubility equilibria.*
- *Examine how complexation affects solubility for the PbI_2 solubility system.*
- *Introduce how the PbI_2 complex will be measured through spectrophotometry in the project lab.*

Brief review of solubility

Textbook links for review references for Chem 103/104 and Chem 109.

The solubility product (K_{sp}) is an equilibrium constant for how much solid is dissolved in aqueous solution. If K_{sp} is large (> 1), the solid nearly completely dissolves. If the solubility product is very small, only a small amount of the solid will dissolve. The solubility product can be written in the form shown below:

$$aA\,(s) \leftrightarrow cD\,(aq) + dD\,(aq) \qquad K_{sp} = [C]^c[D]^d$$

PbI_2 is a common compound used as an example for solubility concepts (K_{sp} = 7.9 x 10^{-9}). It is a bright yellow, sticky powder. In Part 1 of the project lab, you will be exploring the solubility of PbI_2. The following activity will help lead you through concepts that you will see play out in lab.

1. First, let's see what the concentration (in mg/mL) of PbI_2 in H_2O would be in a *saturated solution*. (Hint: You *might* need an ICE table!)

2. In the project lab, you'll be examining the solubility of PbI_2 in H_2O vs hydroiodic acid (HI). Based on what you remember about solubility and Le Châtelier's principle, would you expect more or less PbI_2 to dissolve in HI than water? Explain your reasoning.

What you'll see during the Project Lab is that the opposite is true: more PbI_2 dissolves in HI than H_2O. To understand why, we need to understand how Pb^{2+} exists while in solution. While traditionally we think about ions in a salt separating in solution when they dissolve, some salts are more complicated. They can form what are known as *complexes* in solution. Your textbook actually uses PbI_2 as the example while discussing this phenomenon on pages 134-137 (Harris, Quantitative Chemical Analysis, 10th Edition), Figure 6-3. Each complex has its own equilibrium constant, since the K_{sp} (also an equilibrium constant) cannot account for everything happening in the system. Your textbook denotes these as β_2, β_3, and β_4.

3. Take a moment to talk with your group about what Figure 6-3 is showing. The green line shows a minimum between where Pb solubility is controlled through the common ion effect versus complex formation. At what approximate concentration of iodide does the mechanism of solubility change?

4. In the project lab, you will be working with 57 wt% HI (density = 1.7 g/mL). What is the concentration of HI in molarity? Would using this solvent be in the region where solubility is governed by the common ion effect or complex formation?

In the project lab, you will be measuring the concentration of Pb in solution through absorbance spectroscopy. The various Pb-I complexes absorb light at different wavelengths, as shown in Figure 1. The original figure caption is given along with the citation. Methylammonium iodide (MAI) was an iodide source that was added to a solution containing Pb.

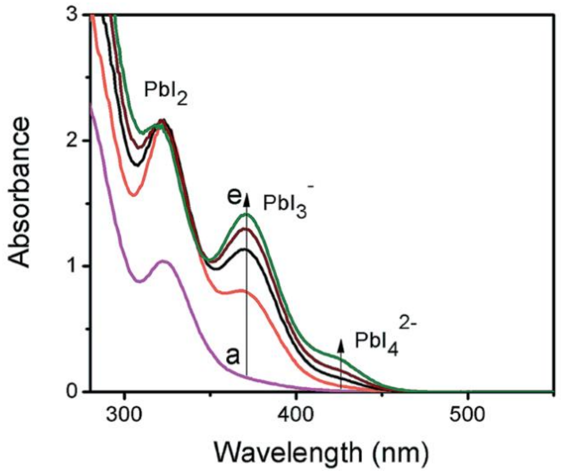

Figure 1. Absorption spectra of 250 mM PbI2 solution in dimethylformamide (a) with increasing concentration of MAI from 6 mM (b) to 24 mM (e). (Reprinted with permission from Stamplecoskie, K. G.; Manser, J. S.; Kamat, P. V. Dual nature of the excited state in organic-inorganic lead halide perovskites. Energy Environ. Sci. ***2015****, 8, 208. Copyright 2015 Royal Chemical Society.)*

5. What do you notice about how the absorbance, and thus the complexation, of Pb-I changes as a source of iodide is added to the system? Does this make sense? Explain your reasoning.

BEGIN PLANNING YOUR PROCEDURE

HINT: You will notice during your experiments that the absorbance traces you collect will likely only have one peak, around 360 nm, indicating the $[PbI_3]^-$ complex. This will let you create a calibration curve under the assumption that all dissolved Pb is in the $[PbI_3]^-$ complex.
