# Supplementary material for: Shining Light on Halide Perovskites: Teaching Analytical Chemistry Using Flexible, Inquiry-Based Experiments
Source: J Chem Educ. 2026 Feb 19;103(3):1480–90. doi: 10.1021/acs.jchemed.5c00906 (PMC12980825; doi:10.1021/acs.jchemed.5c00906)
Supplement: Supplementary file 9 [file ed5c00906_si_014.docx]

Shining Light on Halide Perovskites: Teaching Analytical Chemistry Using Flexible, Inquiry-Based Experiments

Kristel M. Forlano, Eliana Bernat, Pamela Doolittle, Dominic Colosi, Song Jin*, Amanda Rae Buchberger*

Department of Chemistry, University of Wisconsin – Madison, Madison, WI, 53706, United States

*Email:

amanda.buchberger@wisc.edu

jin@chem.wisc.edu

**Preparation for Perovskite Project Lab Part 2**

*Purpose: The following activity is meant to help you think about perovskite crystal structure and semiconductor optical properties as applied to Part 2 of the Perovskite Project Lab. This discussion activity must be completed and submitted to Canvas before you enter lab on the project lab days.*

*Learning Objectives:*

- *Understand the perovskite crystal structure, particularly how the composition can be modified.*
- *Introduce how the perovskite crystal structure impacts the semiconductor properties.*
- *Apply known spectroscopy concepts to a semiconductor material.*

If you haven’t already, watch this video for a good overview of what properties make a semiconductor: <https://www.youtube.com/watch?v=gUmDVe6C-BU>. The other videos linked on the Canvas page are also good overviews of the semiconductor field, and some on perovskites in particular.

In Part 2 of the project lab, you will be making perovskites, a type of semiconductor. Perovskites are crystals, so they have a repeating lattice of connected atoms. The generic crystal structure of a perovskite looks like this:


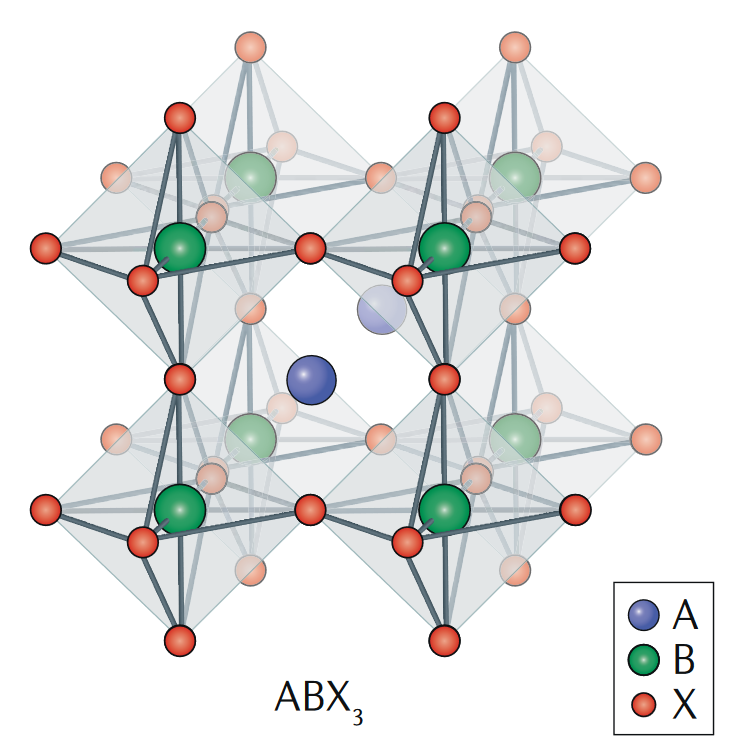

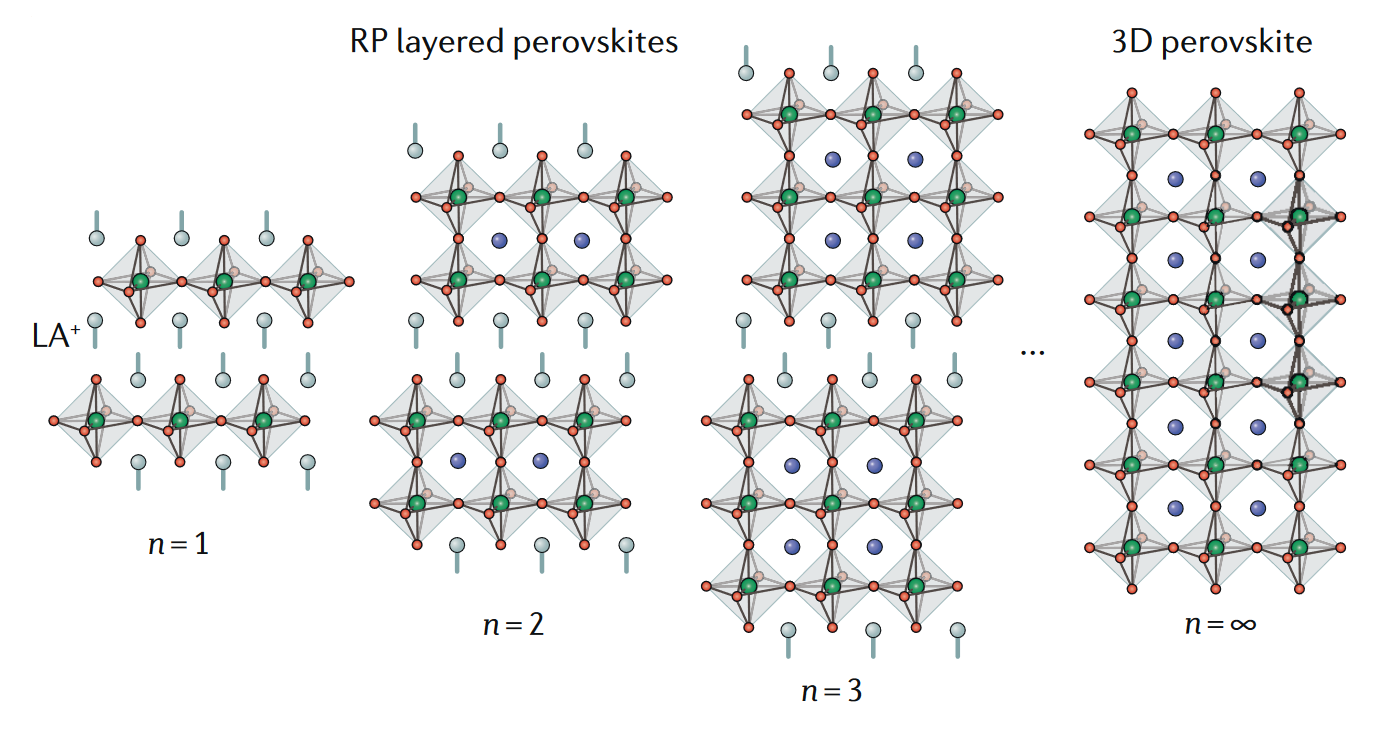


**B)**

**A)**

*Figure 1. A) Example of 3D perovskite crystal structure with formula ABX_3_ and B) Diagram of 2D Ruddlesden-Popper perovskite crystals with formular (LA)_2_A_n-1_B_n_X_3n+1_. Shown for 1 to 3 layers, or n = 1, 2, and 3. The 3D structure is, in essence, n = ∞. (Adapted with permission from Fu et. al.*^1^ *Copyright 2019 Nature.)*

Perovskites are well known for their tunability, or the ability to easily change out the different parts of the structure to get different properties. In the structure shown above in Figure 1A, the “A-site” can be filled with many different small cations, such as cesium (Cs^+^), methylammonium (MA, CH_3_-NH_3_^+^), or formamidinium (FA, NH_2_-CH=NH_2_^+^). The “B-site” can be filled with different, divalent metals, such as lead (Pb^2+^) or tin (Sn^2+^). The “X-site” is filled with a halide, either iodide (I^-^), bromide (Br^-^), or chloride (Cl^-^). 3D perovskites have the formula ABX_3_, so the formula for a perovskite that contains methylammonium, lead, and iodide, would be written as MAPbI_3_.

You will be making what is known as 2D Ruddlesden-Popper (RP) perovskites, shown in Figure 2B. In a 2D perovskite, the 3D crystal structure is “cut” into layers by large organic cations containing an ammonium group. This adds another level of structural tunability through both the choice of spacer cations, as well as how many layers there are of the inorganic octahedra before there is a layer of the spacer cations. 2D RP perovskites have a formula of (LA)_2_(A)*_n_*_-1_(B)*_n_*(X)_3_*_n_*_+1_ where LA is the large ammonium cation and *n* is the number of octahedra layers between cation layers.

1. Write out the formulas for *n* = 1, 2, and 3 perovskite structures using the generic formula (*i.e.,* with LA, A, B, and X as stand-ins).

It is very easy to make perovskite crystals. All that’s needed is to add all the individual components into a suitable solvent. In the lab, you will mostly be given salts of the needed components, for example CsI, as a source of Cs^+^.

1. If you are trying to make an *n* = 1 crystal, why would you be guaranteed to ***not*** make higher *n*?
2. Write out the formula of the *n* = 1 perovskite crystal you would expect to make if you put in hexylammonium iodide (HAI) as your spacer cation (LA), PbI_2_ for your B-site, and have HI as your solvent. *Challenge Question*: Why might hydrohalic acids (HI, HBr, *etc.*) be a particularly good solvent for making perovskites? *(Think back to Part 1 and more….)*

If perovskites can be made by combining the different components together, how can the final aspect of tunability, the *n* number, be controlled? The easy answer is stoichiometry. Theoretically, if the stoichiometric amounts of each precursor were placed in solution for a certain *n* number crystal, then that’s what crystal should form. Unfortunately, reality isn’t quite so nice, and many times there needs to be a lot of fine tuning of the recipes to get the crystals wanted instead of a different crystal or a mix of crystal types. However, we can have stoichiometry as a starting point and use Le Chatelier’s principle to help guide us on how to change our recipe.

1. If you have an *n* = 2 perovskite of (PEA)_2_(MA)Pb_2_I_7_ (PEA = phenethylammonium, MA = methylammonium) and want to instead make the *n* = 3 crystal, how should you change your recipe? *Hint:* Write out the *n* = 3 formula and take note of how it’s different from *n* = 2.

The tunability of the perovskite crystal structure is very important due to how the bandgap energy is affected by the different components. In perovskites, the semiconductor nature is formed due to the orbital overlap of the B-site and X-site atoms. Therefore, changing lead to tin in the B-site or bromide to iodide in the X-site can have a large effect. The other major way that the bandgap energy of perovskites can be modified is through the *n* number. When an electron is excited in a semiconductor to the conduction band, it leaves an area of positive charge behind, aptly named a “hole”. Because of the negative and positive charges of the electron and hole respectively, these two charges are semi bound together as a quasi-particle called an exciton. However, excitons want to exist within a certain real space. Whenever a material is so small that the exciton cannot occupy the space that it would like to, it is said to be “quantum confined”. Quantum confinement in a material can have drastic effects on the semiconductor properties, such as in quantum dots, which are semiconductor nanoparticles, that won the noble prize in chemistry for their discovery in 2023 (<https://www.nobelprize.org/prizes/chemistry/2023/press-release/>).

The quantum confinement effect in perovskites comes from the 2D nature of the layers, shown in Figure 2A. Between the quantum confinement effects and tunable composition, the bandgap energies of 2D perovskites can vary widely, as shown through the varied photoluminescent emissions in Figure 2B.


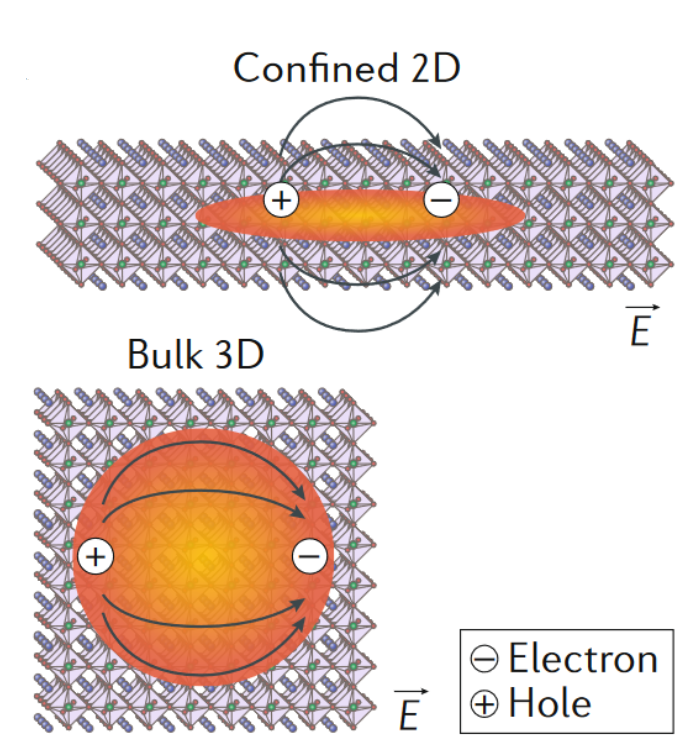

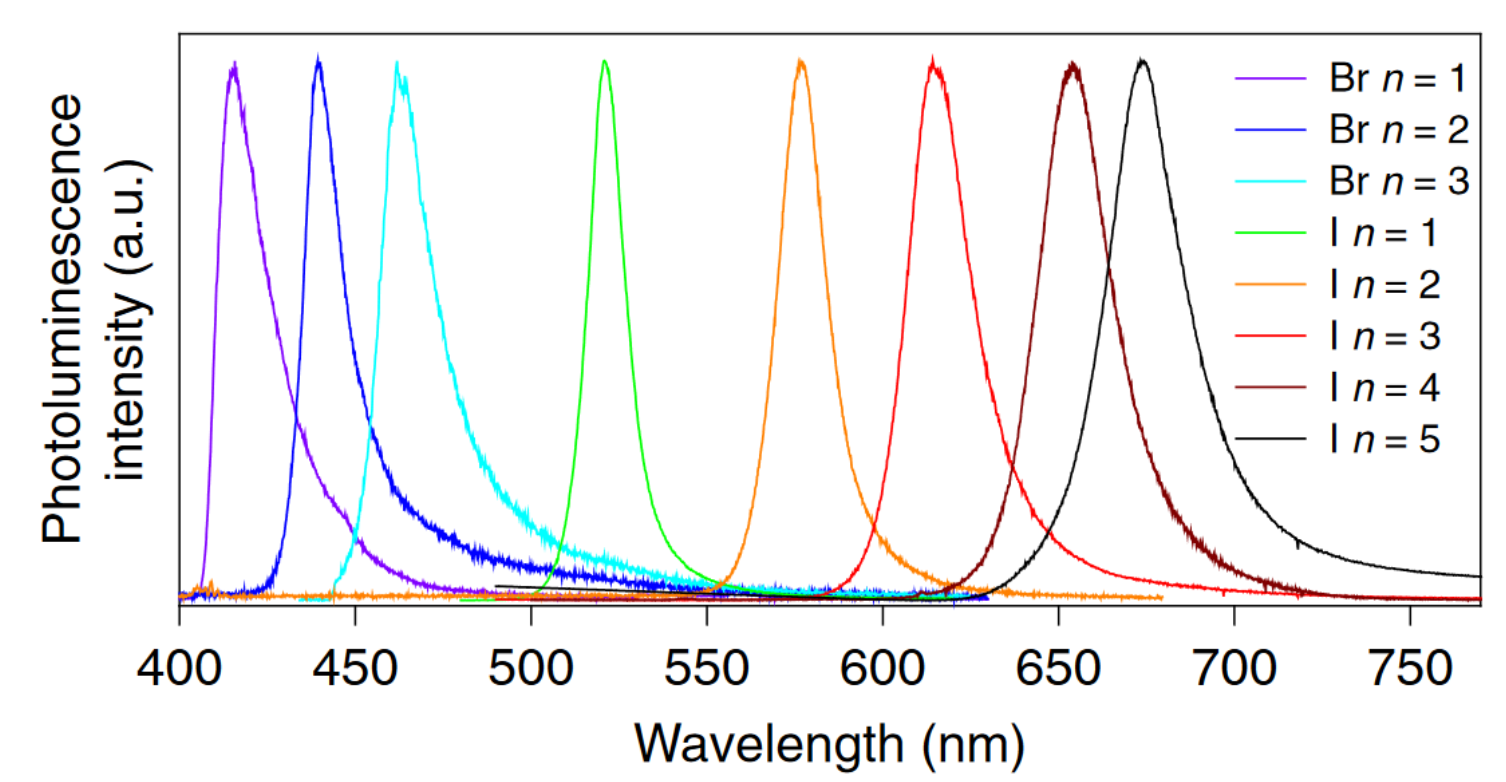


*Figure 2. A) Visual of quantum confinement of an exciton in 2D perovskites vs not quantum confined in 3D perovskites. (Adapted with permission from Fu et. al.*^1^ *Copyright 2019 Nature.) and B) Photoluminescent emission for 2D RP perovskites with various compositions (Adapted with permission from Pan et. al.*^2^ *Copyright 2021 Nature.).*

In Part 2, you will be measuring the optical properties of the perovskite crystals, including their transmittance and photoluminescence. You are likely used to measuring optical properties by using a cuvette filled with a solution. But, how do you measure a crystal? There are some spectrometer set-ups that allow you to measure individual crystals, but we do not have access to this type of equipment. Instead, you will be measuring the crystals by making thin films or thin film-like samples and figuring out how to modify the way you take measurements with a cuvette-based spectrometer. Your lab manual will walk you through this process. However, there are some important concepts to think through with these measurements.

1. The bandgap energy of semiconductors is regularly expressed in electron volts (eV). Light is also energy, and therefore we can figure out what wavelength of light (in nm) corresponds to the energy of the bandgap through the equation *E = (h*c)/λ*, where *h* is Planck’s constant and *c* is the speed of light. If an *n* = 1 lead iodide perovskite has a bandgap energy of 2.35 eV, what wavelength does this correspond to? (1eV = 1.60 x 10^-19^ J)
2. The bandgap is the difference energetically between the valence and conduction band energy levels. To create an exciton, or the electron-hole pair created by exciting an electron to the conduction band, do you need more, the same, or less energy than the bandgap energy? Sketch what you’d expect for the absorption spectrum of an *n* = 1 lead iodide perovskite, using the information from question 5.


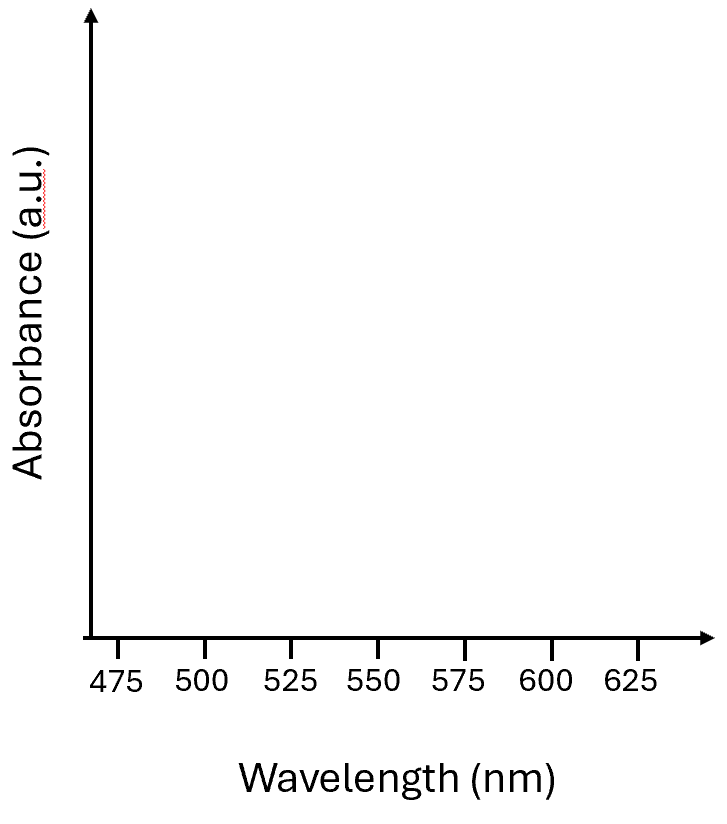


1. Perovskite crystals are also photoluminescent. What is this a result of? How is the photoluminescence and the bandgap energy related? It could help to draw a Jablonski diagram of the processes.
2. You *will not* be able to measure absorbance directly with the perovskite crystals. Instead, you can only measure transmittance. If you set your spectrometer to read the absorbance, it will read the sample incorrectly as these spectrometers calculate absorbance through *A = -log(T)*. Why can you not measure absorbance directly? Hint: Think of all the ways light may interact with a sample, particularly shiny crystals.
3. Since you’ll be measuring transmittance instead of absorbance, sketch what you think a transmittance curve will look like.


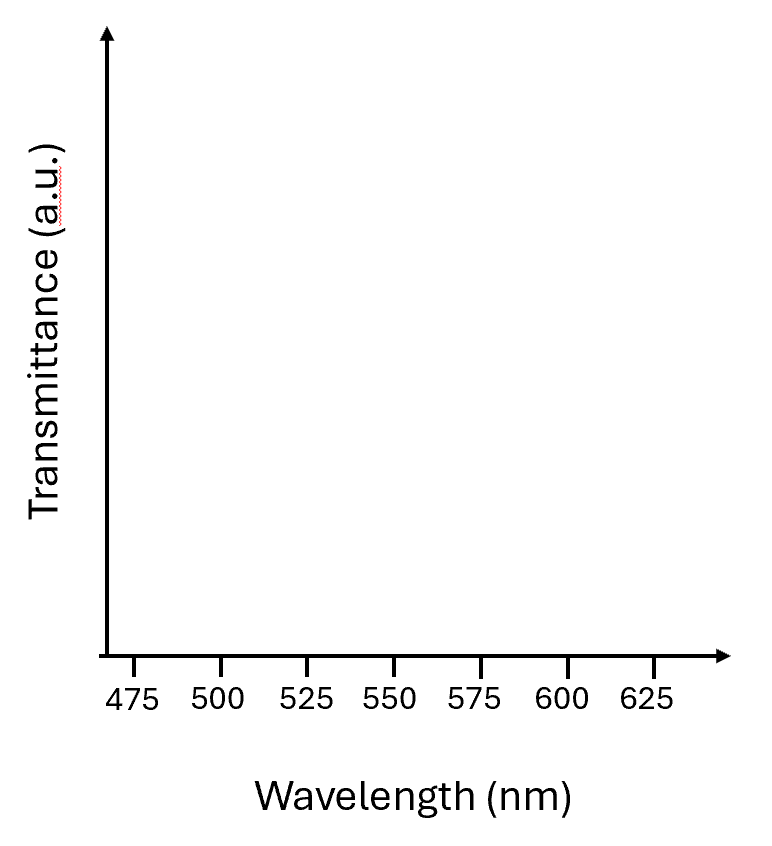


If you’ve reached the end, go check out some more of the videos linked on Canvas. If any of the material is confusing, hearing it explained multiple ways, with different visuals, can be helpful!

References:

(1) Fu, Y.; Zhu, H.; Chen, J.; Hautzinger, M. P.; Zhu, X.-Y.; Jin, S. Metal Halide Perovskite Nanostructures for Optoelectronic Applications and the Study of Physical Properties. *Nat Rev Mater* **2019**, *4* (3), 169–188. https://doi.org/10.1038/s41578-019-0080-9.

(2) Pan, D.; Fu, Y.; Spitha, N.; Zhao, Y.; Roy, C. R.; Morrow, D. J.; Kohler, D. D.; Wright, J. C.; Jin, S. Deterministic Fabrication of Arbitrary Vertical Heterostructures of Two-Dimensional Ruddlesden–Popper Halide Perovskites. *Nat. Nanotechnol.* **2021**, *16* (2), 159–165. https://doi.org/10.1038/s41565-020-00802-2.
